# Utilizing Deep Learning Algorithms for Signal Processing in Electrochemical Biosensors: From Data Augmentation to Detection and Quantification of Chemicals of Interest

**DOI:** 10.3390/bioengineering10121348

**Published:** 2023-11-23

**Authors:** Fatemeh Esmaeili, Erica Cassie, Hong Phan T. Nguyen, Natalie O. V. Plank, Charles P. Unsworth, Alan Wang

**Affiliations:** 1 Department of Engineering Science, University of Auckland, Auckland 1010, New Zealand; fesm704@aucklanduni.ac.nz (F.E.); c.unsworth@auckland.ac.nz (C.P.U.); 2 The MacDiarmid Institute for Advanced Materials and Nanotechnology, Victoria University of Wellington, Wellington 6021, New Zealand; erica.cassie@vuw.ac.nz (E.C.); jenna.nguyen@vuw.ac.nz (H.P.T.N.); natalie.plank@vuw.ac.nz (N.O.V.P.); 3 School of Chemical and Physical Sciences, Victoria University of Wellington, Wellington 6021, New Zealand; 4 Auckland Bioengineering Institute, University of Auckland, Auckland 1010, New Zealand; 5 Center for Medical Imaging, Faculty of Medical and Health Sciences, University of Auckland, Auckland 1010, New Zealand; 6 Centre for Brain Research, University of Auckland, Auckland 1010, New Zealand

**Keywords:** data augmentation, conditional variational auto-encoder (CVAE), signal extrapolation, convolutional neural network (CNN), convolutional long short-term memory (ConvLSTM), long short-term memory (LSTM), gated recurrent unit (GRU), deep learning classification

## Abstract

Nanomaterial-based aptasensors serve as useful instruments for detecting small biological entities. This work utilizes data gathered from three electrochemical aptamer-based sensors varying in receptors, analytes of interest, and lengths of signals. Our ultimate objective was the automatic detection and quantification of target analytes from a segment of the signal recorded by these sensors. Initially, we proposed a data augmentation method using conditional variational autoencoders to address data scarcity. Secondly, we employed recurrent-based networks for signal extrapolation, ensuring uniform signal lengths. In the third step, we developed seven deep learning classification models (GRU, unidirectional LSTM (ULSTM), bidirectional LSTM (BLSTM), ConvGRU, ConvULSTM, ConvBLSTM, and CNN) to identify and quantify specific analyte concentrations for six distinct classes, ranging from the absence of analyte to 10 μM. Finally, the second classification model was created to distinguish between abnormal and normal data segments, detect the presence or absence of analytes in the sample, and, if detected, identify the specific analyte and quantify its concentration. Evaluating the time series forecasting showed that the GRU-based network outperformed two other ULSTM and BLSTM networks. Regarding classification models, it turned out signal extrapolation was not effective in improving the classification performance. Comparing the role of the network architectures in classification performance, the result showed that hybrid networks, including both convolutional and recurrent layers and CNN networks, achieved 82% to 99% accuracy across all three datasets. Utilizing short-term Fourier transform (STFT) as the preprocessing technique improved the performance of all datasets with accuracies from 84% to 99%. These findings underscore the effectiveness of suitable data preprocessing methods in enhancing neural network performance, enabling automatic analyte identification and quantification from electrochemical aptasensor signals.

## 1. Introduction

Deep learning algorithms have gained attention in the context of sensor development and application. These algorithms have been effective and beneficial for addressing problems such as noise reduction, classification, object detection, anomaly detection, and process monitoring [1,2,3].

There has been a wide range of deep learning models for classification, such as convolutional neural network (CNN), generative adversarial network (GAN)-based architectures [4], and recurrent-based neural networks [5,6]. For example, Zhang [7] proposed a CNN-based network to automatically identify and quantify heavy-metal ions, which obtained with an accuracy of 99.99%. Moreover, Li [8] proposed a hybrid network, utilizing convolutional and long short-term memory layers in the network architectures, for EEG signal classification to find Parkinson’s disease. The classification accuracy for this model was 98.6%.

Moreover, deep learning-based generative models, such as variational autoencoders (VAEs), generative adversarial networks (GANs), and diffusion probabilistic models, have found extensive use in data augmentation [9]. Specifically, variational autoencoders (VAEs) and their extension, conditional variational autoencoders (CVAE), have been applied in research for anomaly detection, generating new sample data, and reconstructing input data by learning the fundamental features and structure of the training data. For instance, Liu [10] utilized VAE and CVAE structures for data augmentation in an intrusion detection system (IDS) to address data imbalances. The application of these networks significantly improved model performance metrics, specifically the Macro F1-score, by 5.32%. Moreover, CVAE proved to be effective in detecting structural anomalies in scanning transmission electron microscopy (STEM) images. By accurately reproducing input data and highlighting discrepancies in defect input data, CVAE was capable of automatically differentiating various point defect types [11].

Furthermore, deep learning networks have emerged as powerful tools for time series forecasting or predicting future data, using techniques such as CNNs, recurrent neural networks (RNNs), and temporal convolutional networks (TCNs) [12]. For example, Pirani [13] explored various RNN-based architectures for financial time series forecasting, demonstrating that models incorporating gated recurrent unit (GRU) layers outperformed other recurrent networks. Likewise, Mahjob [14] used quite similar architectures for energy consumption prediction, and the results showed that the network containing long short-term memory (LSTM) layer in its architectures outperformed other RNN-based models.

The motivation for this paper was related to our previous work [15,16]. In the former, we used LSTM-based networks for classification. We aimed to enhance the robustness, performance and speed of the automatic detection and quantification of analyte concentrations registered by sensors. Also, the results of both studies raised an issue that the time for recording the signals might have been effective in the outcomes of the deep learning models. Thus, we were inspired to employ techniques to forecast the future output of the sensors and examine our hypothesis about the signals’ lengths in the sensors’ signal analysis.

Thus, it can be briefly said that, in this study, we applied deep learning algorithms for signal processing obtained from three comparable CNT FET biosensors. Our primary objective was to automatically identify and quantify specific analytes using segments of these recorded signals. However, achieving this classification objective posed challenges that needed to be addressed. Consequently, we employed three key deep learning-based signal analysis techniques to overcome these challenges and fulfil our research objectives. These techniques were categorized into three main steps: (1) data augmentation, (2) signal extrapolation, and (3) classification models. Additionally, all calculations and deep learning algorithms were executed using MATLAB R2022b.

**Figure 1:**
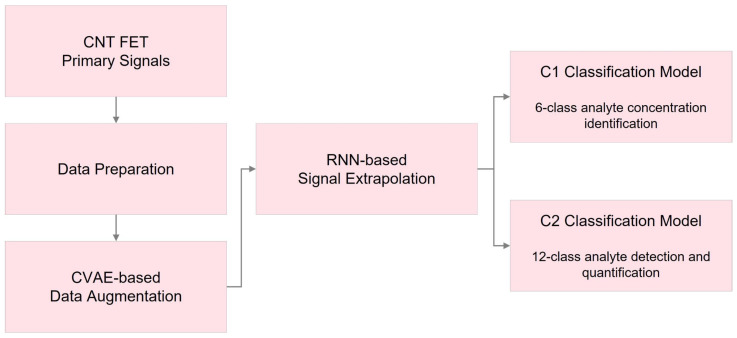
Workflow of the proposed deep learning techniques in this study.

Figure 1 illustrates the framework of the applied deep learning methods in this research. Initially, essential data preparation steps were applied to the available datasets, including z-score scaling and STFT. Subsequently, a CVAE-based data augmentation technique was used to handle the limited data availability. In the following step, a signal extrapolation method was employed, utilizing recurrent neural networks (RNN) to generate uniform signal lengths for all the datasets.

Finally, we designed two classification models, C1 and C2, incorporating recurrent and convolutional layers. The C1 model aimed to identify and measure precise analyte levels within six distinct categories, ranging from no analyte presence to 10 μM. Each dataset’s segments were analyzed separately in this model. The C2 classification model, on the other hand, was developed to differentiate abnormal data from normal segments, identify the absence or presence of analytes in the sample, and, if present, recognize the specific substance and measure its concentration.

## 2. Background of Deep Learning Models

### 2.1. Recurrent Neural Network

Recurrent Neural Networks (RNNs) [17,18] are a specialized type of feed-forward neural network designed for processing sequential data. An RNN consists of hidden recurrent units, where the output at a specific time step is calculated based on the output from the previous time step.

**Figure 2:**
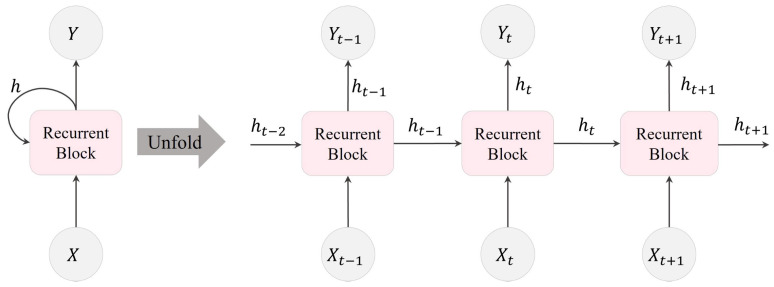
Visualization of an RNN unfolded in time, illustrating the transmission of information within an RNN layer over three consecutive time steps.

In Figure 2, the movement of information within an RNN layer across three consecutive time steps is illustrated. Here, $X\in R^{N}$, $h\in R^{L}$, and $Y\in R^{M}$ represent the input to the RNN layer, the output of the RNN layer, and the subsequent layer after the RNN, respectively. A recurrent block comprises multiple recurrent units within the RNN layer. In this depiction, the number of hidden recurrent units, denoted as *L*, is equivalent to the dimension of *h*.

In the following, we will delve into the RNN structures utilized in this study. We will begin by exploring the basic structure of an RNN, followed by its two main extensions: GRU and LSTM.

#### 2.1.1. Conventional Recurrent Neural Network

**Figure 3:**
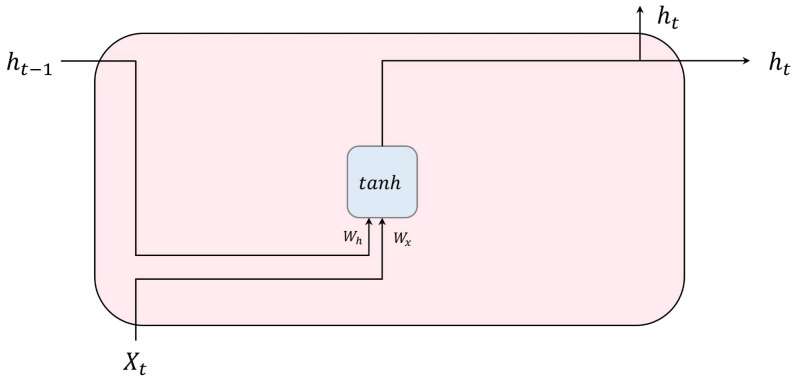
Structure of a vanilla RNN unit using a hyperbolic tangent function as its activation function.

The vanilla RNN, also known as the conventional RNN, represents the most basic form of recurrent neural networks, as shown in Figure 3. It undergoes training utilizing the backpropagation through time technique [17]. In this model, an input sequence $X_{t}\in R^{N}$ is fed into a recurrent unit at time step *t*. The output $h_{t}$ of the unit at the time step *t* is calculated using Equation (1):

(1) $$h_{t}=\left\{\begin{array}{cc} 0 & t=0,\\ \phi (W_{x}\;X_{t}+W_{h}\;h_{t-1}+b) & otherwise, \end{array}\right.$$

where ϕ represents an activation function, $W_{x}$, $W_{h}$, and *b* indicate weight matrices and the bias vector, respectively.

Vanilla RNNs, while powerful dynamic systems, face challenges in training due to the vanishing gradient problem during backpropagation. This problem arises because the backpropagated gradients tend to either shrink or grow at each time step, leading to vanishing or exploding gradients over many time steps. Consequently, vanilla RNNs struggle to capture long-term dependencies. To address this issue, two main extensions of RNNs were developed: (i) LSTM, and (ii) GRU networks. These extensions integrate gating mechanisms within their recurrent units, resolving the issue of vanishing gradients and facilitating the capture of long-term dependencies.

#### 2.1.2. Long Short-Term Memory

**Figure 4:**
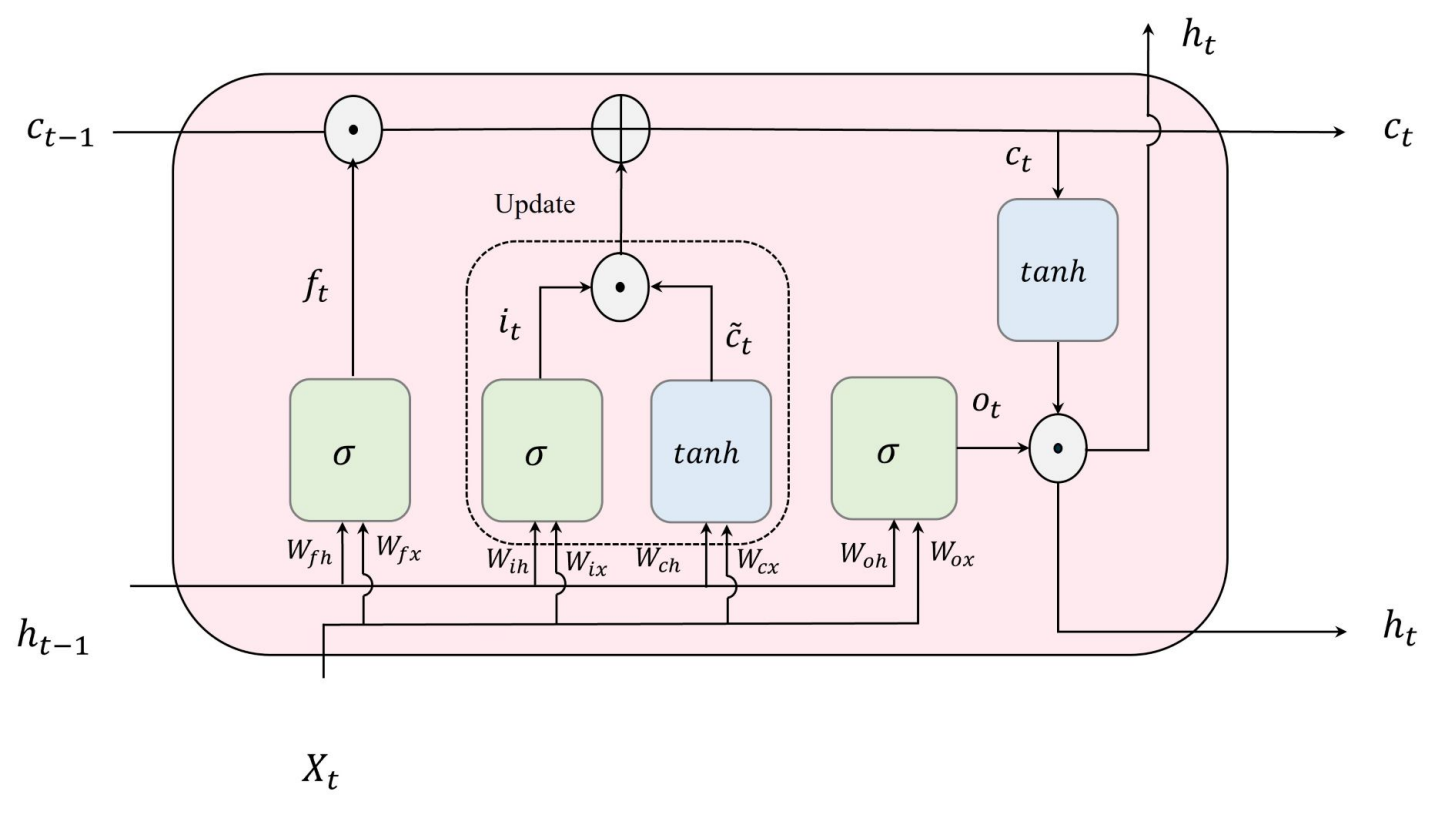
Structure of an LSTM unit or an LSTM hidden unit (⊕ and ⊙ refer to element-wise addition and multiplication, respectively).

Long Short-Term Memory or LSTM [19], an advanced extension of RNNs, excels in learning temporal dependencies within sequential input during the training phase due to the unique structure of its recurrent units, known as LSTM cells. These cells incorporate three key components: the input gate, forget gate, and output gate, as depicted in Figure 4. These gates play a vital role in regulating the transmission of information entering and exiting the unit, enabling the LSTM network to capture temporal dependency in sequential data.

The input gate, represented as $i_{t}$, and the cell candidate, denoted as ${\tilde{c}}_{t}$, are in charge of modifying and managing the memory cell state, $c_{t}$. The forget gate, denoted as $f_{t}$, decides which details from the previous time step t−1 stored in the cell state $c_{t-1}$ should be disregarded. The output gate, $o_{t}$, selects the portion of the cell state that contributes to the output, $h_{t}$. Equations (2)–(6) governing these processes at time step *t* are as follows:

(2) $$i_{t}=\sigma \left(W_{ih}\;h_{t-1}+W_{ix}\;X_{t}+b_{i}\right),$$

(3) $$f_{t}=\sigma \left(W_{fh}\;h_{t-1}+W_{fx}\;X_{t}+b_{f}\right).$$

(4) $${\tilde{c}}_{t}=tanh\left(W_{ch}\;h_{t-1}+W_{cx}\;X_{t}+b_{c}\right),$$

(5) $$c_{t}=i_{t}\times {\tilde{c}}_{t}+f_{t}\times c_{t-1},$$

(6) $$o_{t}=\sigma \left(W_{oh}\;h_{t-1}+W_{ox}\;X_{t}+b_{o}\right),$$

(7) $$h_{t}=o_{t}\times tanh\left(c_{t}\right),$$

where $W_{ix}$, $W_{fx}$, $W_{cx}$, and $W_{ox}$ denote the input gate’s weight matrices for input, forget, cell value, and output, respectively. Similarly, $W_{ih}$, $W_{fh}$, $W_{ch}$, and $W_{oh}$ represent the recurrent gate weights for input, forget, cell value, and output, respectively. In the same sequence, the corresponding bias vectors are denoted as $b_{i}$, $b_{f}$, $b_{c}$, and $b_{o}$.

**Figure 5:**
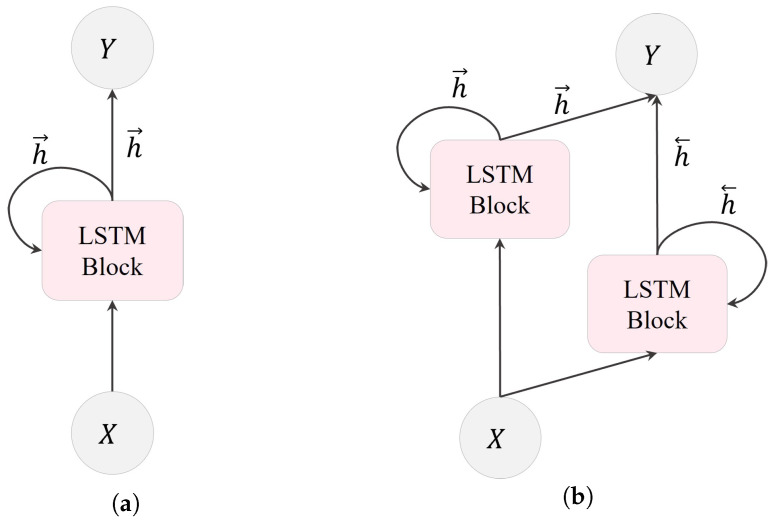
Transmission of data within an LSTM layer: (**a**) a ULSTM layer with forward states, and (**b**) a BLSTM layer with both forward and backward states. Here, *X*, *Y*, $\vec{h}$, and $\overset{\leftarrow }{h}$ represent the input, output, forward and backward states in the BLSTM layer, respectively.

In an LSTM layer, multiple LSTM units are recurrently connected. As shown in Figure 5, there are two types of LSTM layers: unidirectional LSTM (ULSTM) and bidirectional LSTM (BLSTM). These categories are based on the information flow within the layer. ULSTM processes information in one direction, moving forward in time, as illustrated in Figure 5a. Conversely, BLSTM, depicted in Figure 5b, consists of both forward and backward layers. The forward layer operates in the forward time direction, whereas the backward layer handles information processing in the reverse time direction.

#### 2.1.3. Gated Recurrent Unit

**Figure 6:**
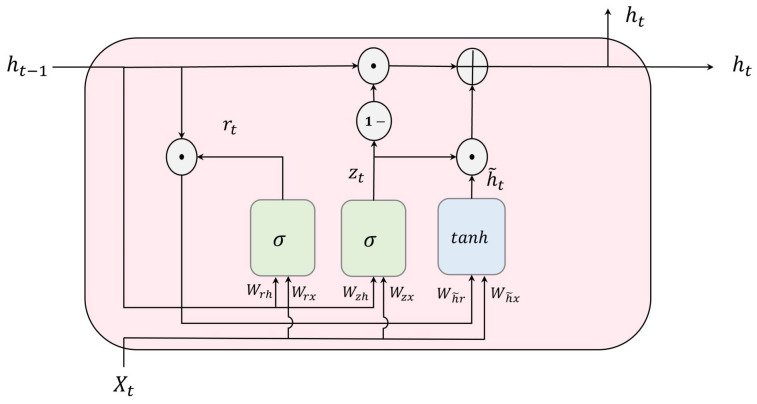
Illustration of GRU structure (⊕ and ⊙ refer to element-wise addition and multiplication, respectively).

A Gated Recurrent Unit or GRU network is an extension of RNNs and a simplified variant of LSTM, lacking an output gate [20,21,22]. In comparison to LSTM, GRU is more straightforward and faster during training. Unlike LSTM, GRU does not utilize a memory cell state to retain information. Instead, it only controls information within the unit. Unlike LSTMs, GRUs fully expose the memory in each state. The GRU architecture comprises three key components: the update gate, the reset gate, and a candidate state, as illustrated in Figure 6.

The update gate, $z_{t}$, determines the proportion of the previous memory that should be retained during the training process, while the reset gate, $r_{t}$, decides how much of the new input and the previous memory to combine. The following equations mathematically depict the calculation of the output $h_{t}$ at time step *t*.

(8) $$z_{t}=\sigma \left(W_{zx}\;X_{t}+W_{zh}\;h_{t-1}+b_{z}\right),$$

(9) $$r_{t}=\sigma \left(W_{rx}\;X_{t}+W_{rh}\;h_{t-1}+b_{r}\right),$$

(10) $${\tilde{h}}_{t}=tanh\;\left(W_{\tilde{h}x}\;X_{t}+W_{\tilde{h}r}\;\left(r_{t}⊙h_{t-1}\right)+b_{\tilde{h}}\right),$$

(11) $$h_{t}=\left(1\;-z_{t}\right)\;⊙\;h_{t-1}+\;z_{t}\;⊙\;{\tilde{h}}_{t},$$

where $X_{t}$ is the input vector, $z_{t}$ is the update gate, $r_{t}$ is the reset gate, ${\tilde{h}}_{t}$ is the candidate state, and σ is the sigmoid activation function. *W* and *b* refer to the weights matrices and bias vectors, respectively.

### 2.2. Convolutional Neural Network

**Figure 7:**
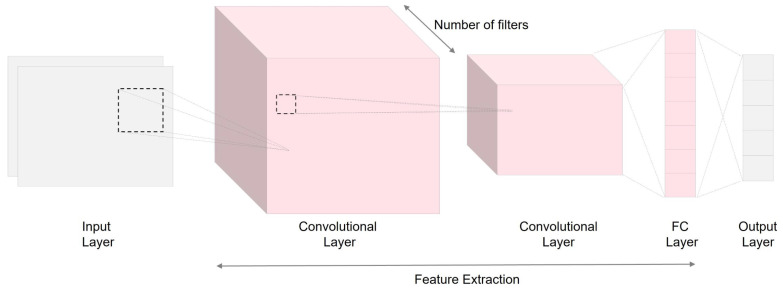
A typical structure of CNN.

Convolutional neural networks (CNN) are designed to analyze data presented in 1D, 2D, or 3D arrays, such as signals, images, or videos. CNNs possess four essential characteristics: local connections, where neurons focus on specific regions; shared weights, enabling parameter efficiency; pooling, reducing spatial dimensions; and the incorporation of multiple layers, allowing the network to extract complicated features from the data. These characteristics optimize CNNs for processing array data effectively [23]. As shown in Figure 7, the typical composition of a CNN includes fundamental layers like convolutional, pooling, and fully connected layers.

Convolutional layers process multiple input feature maps using filters to generate output feature maps. This operation involves sliding the filter across the entire input array. Each neuron calculates a weighted sum in a convolutional layer based on a subset of the prior layer’s outputs [24]. Consider $X_{i,j}$ as the prior layer’s output, where *i* and *j* denote the specific position in the two-dimensional grid. The weights assigned to an individual node within the present layer are denoted as $w_{k,l}$, with *k* and *l* indicating the respective weight indices. As represented in Equation  (12), the calculated 2D convolution or the sum of weights, can be written as follows:

(12) $$Y_{i,j}=\sum_{k}\sum_{l}w_{k,l}\cdot X_{i,j}.$$

It needs to be mentioned that the output size of a convolution layer, $l_{n}$, by the padding *p*, stride *s*, is calculated according to Equation (13).

(13) $$l_{n}=\left[\frac{l_{n-1}+2p-n_{f}}{s}+1\right],$$

where $l_{n-1}$ and $n_{f}$ indicate the previous layer size and number of filters, respectively.

Following convolutional layers, the output neurons are fed into a non-linear activation function, such as the Rectified Linear Unit (ReLU) or Leaky ReLU, illustrated in Equations (14) and (15), respectively.

(14) f(x)=max(0,x).

(15) $$f\left(x\right)=\left\{\begin{array}{cc} x & x\geq 0,\\ a\cdot x & x<0, \end{array}\right.$$

where *a*, the slope coefficient or threshold, is a small positive constant that multiplies the negative input values, ensuring a small gradient for negative inputs, allowing some information to flow even for negative inputs.

### 2.3. Variational Autoencoder

**Figure 8:**
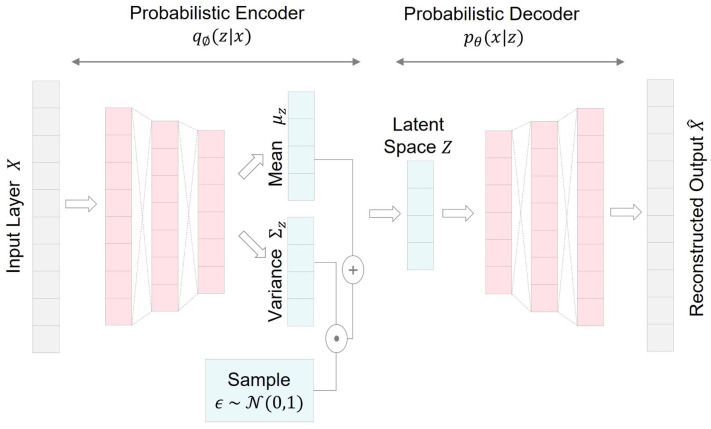
Ilustration of a variational autoencoder (VAE) network architecture.

A variational autoencoder (VAE) is a generative learning model developed from the Bayesian framework, as introduced in [25]. A VAE comprises a recognition model, also known as a variational approximator, denoted as $q_{\phi }\left(z|x\right)$ and a generative model $p_{\theta }\left(z\right)p_{\theta }\left(x|z\right)$, where *x* and *z* represent input data and latent space, respectively. The architecture of a VAE network is depicted in Figure 8.

The objective of the VAE is to jointly learn the parameters of variational approximator ϕ and the generative model θ. This is achieved by maximizing the evidence lower bound (ELBO), defined in Equation (16):

(16) $$L_{\mathrm{vae}}\left(\theta ,\phi ;x\right)=-D_{KL}(q_{\phi }\left(z|x\right)||p_{\theta }\left(z\right))+E_{q_{\phi }\left(z|x\right)}\left[\log p_{\theta }\left(x|z\right)\right],$$

where $D_{KL}$ denotes the Kullback–Leibler (KL) divergence between the approximated posterior $q_{\phi }\left(z|x\right)$ and the prior of the latent variable $p_{\theta }\left(z\right)$. The first term on the right-hand side acts as a regularizer, encouraging minimising the KL divergence between $q_{\phi }\left(z|x\right)$ and $p_{\theta }\left(z\right)$. The second term represents an expected negative reconstruction error, maximizing the log-likelihood $logp_{\theta }\left(x|z\right)$ with samples drawn from the approximated posterior.

To efficiently estimate this optimization, the stochastic gradient variational Bayes (SGVB) method, employing the autoencoding variational Bayes (AEVB) algorithm and a reparameterization trick [25], is used. The reparameterization trick involves reparameterizing the approximated posterior using a differentiable transformation $g_{\phi }\left(\epsilon ,x\right)$, where ϵ is a random noise variable acting as an auxiliary variable with an independent marginal p(ϵ). This trick overcomes the challenge of calculating the gradient of $L_{\mathrm{vae}}$ with respect to ϕ.

In practical applications, the choice of probability distributions is pivotal. Typically, a Gaussian distribution $N(\mu_{z},\Sigma_{z})$ is selected for the posterior, where μ and Σ are arbitrary deterministic functions [26]. For the prior, a standard normal distribution N(0,1) is commonly chosen. Regarding the likelihood distribution, a multivariate Gaussian distribution is used for continuous data, while a Bernoulli distribution is suitable for binary data [27].

### 2.4. Conditional Variational Autoencoder

**Figure 9:**
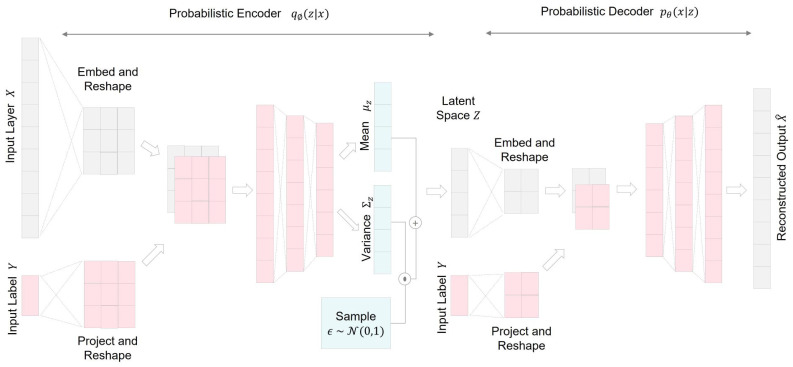
Illustration of conditional variational autoencoder (CVAE) network architecture that integrates conditional information for enhanced generative modeling.

The conditional variational autoencoder (CVAE) extends the capabilities of the VAE by incorporating external information, termed conditional data, during the generative process. Introduced in [28], the CVAE addresses a limitation inherent in VAEs. Traditional VAEs can generate data solely from latent variables, representing a specific class of sample data. In contrast, as depicted in Figure 9, a CVAE can create data using both latent variables and additional conditional input, allowing it to generate sample data for a specific class based on corresponding class labels [10]. While VAEs are primarily considered an unsupervised training framework, CVAEs operate in a semisupervised manner as their inputs include both sample data and class labels.

Equation (17) represents the objective function of the CVAE, which is similar to that of the VAE but includes additional data class label information:

(17) $$L_{\mathrm{cvae}}\left(\theta ,\phi ;x\right)=-D_{KL}(q_{\phi }\left(z|x,y\right)||p_{\theta }\left(z\right))+E_{q_{\phi }\left(z|x\right)}\left[\log p_{\theta }\left(x|z,y\right)\right],$$

where *y* refers to the class label.

## 3. Materials

This section gives a summary of the datasets used in this study, the sensing protocols for their corresponding signal registration, along with a detailed explanation of the data preparation and preprocessing techniques applied. A comprehensive understanding of the datasets, their preparation, and preprocessing is crucial for gaining insights into the subsequent classification models.

### 3.1. Datasets Description

A dataset of sensors, represented as $D=\{X_{1},\ldots ,X_{s},\ldots ,X_{S}\}$, consists of a collection of signals denoted as $X_{s}$, which are registered from identical sensors. Here, the subscript *s* is the index of each signal, while *S* signifies the total count of signals within this dataset.

In this study, we used and analyzed three distinct datasets: (1) 35-mer adenosine, (2) 31-mer oestradiol, and (3) 35-mer oestradiol. These datasets include multiple single-variable time-series signals that record the drain current from three distinct sensors. Specifically, the sensors were based on technology utilizing aptamer-based electrochemical carbon nanotube field-effect transistors (CNT-FETs).

Table 1 presents the available datasets and their sizes, including the total number of signals in each dataset. In order to enhance comprehension of the registered signals, a brief introduction to the sensors and the sensing protocols used to measure drain current is provided below, outlining the components of the sensors and the methodology used for signal measurement.

Table 2 represents three fundamental components of the sensors employed in collecting data to concisely compare the datasets. The datasets were primarily differentiated by two key elements of their sensors: (i) their target analytes and (ii) their aptamers employed as the bioreceptors. The other key component, which is the transducer, was CNT FET for all three sensors. Providing a comprehensive explanation of the sensor functionalization details is outside the scope of this paper. Nevertheless, comprehensive details regarding the 35-mer adenosine sensor, including specifics about receptor functionalization and transistor fabrication, are available, in [29].

In terms of sensing protocols, the procedures for the 31-mer and 35-mer oestradiol sensors shared similarities, but they differed from those employed for the adenosine sensors. Table 3 compares and summarizes the sensing procedures used in the datasets.

The signals for oestradiol and adenosine were registered at specific time intervals, with oestradiol measured every 1.081 with a standard deviation of $5\times 10^{-3}$ and adenosine every 1 s, respectively. The drain and gate voltages were held consistently at $V_{D}=100$ mV and $V_{G}=0$ V for both.

Initially, the polydimethylsiloxane (PDMS) wells of the sensors were filled with designated solutions. The adenosine sensor employed a 2 mM Tris-HCl buffer, while the oestradiol sensors utilized a solution consisting of 0.05 times PBS (phosphate-buffered saline) with 5% ethanol (EtOH).

In the case of the adenosine aptasensor, the process commenced by filling the PDMS well with 110 μM of 2 mM Tris-HCl, which was maintained for 1000 s. Following this, adenosine solution was introduced into the PDMS well every 500 s, with each injection containing progressively higher concentrations, taking into account the total adenosine concentration in the well before each addition. Through this incremental approach, the adenosine concentration in the well was raised, ranging from 100 pM to 10 μM.

Considering the oestradiol aptasensors, the procedure started by introducing an initial volume of 100 µL of 0.05 times PBS with 5% EtOH into the PDMS well, which was maintained for 300 s. During the subsequent 300 s, an additional 20 μL of 0.05 times PBS with 5% EtOH was added without altering the oestradiol concentration. Afterward, oestradiol solution was injected into the well every 300 s, taking into account the existing oestradiol concentration before each addition. Furthermore, with each increment in the oestradiol concentration, 20 µL of 0.05 times PBS with 5% EtOH was introduced into the well. This step-by-step process elevated the oestradiol concentration within the well, ranging from 1 nM to 10 μM.

**Figure 10:**
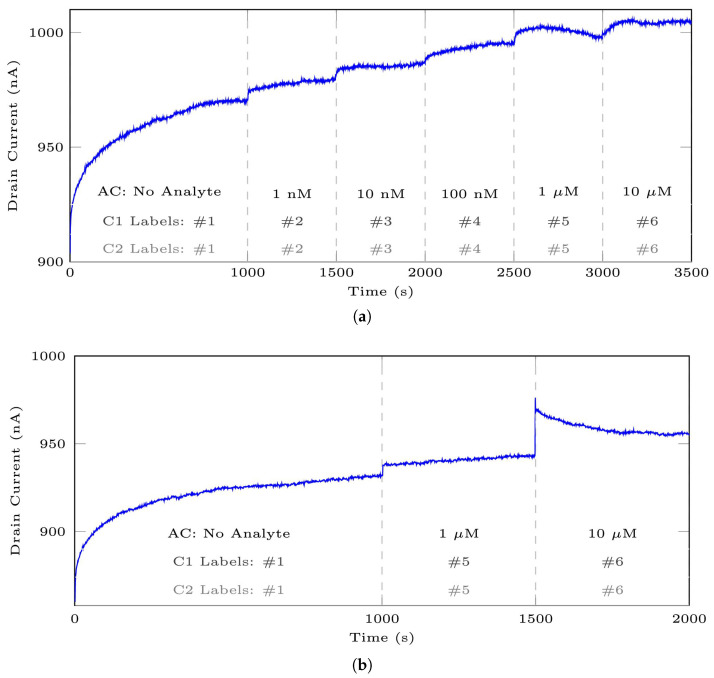

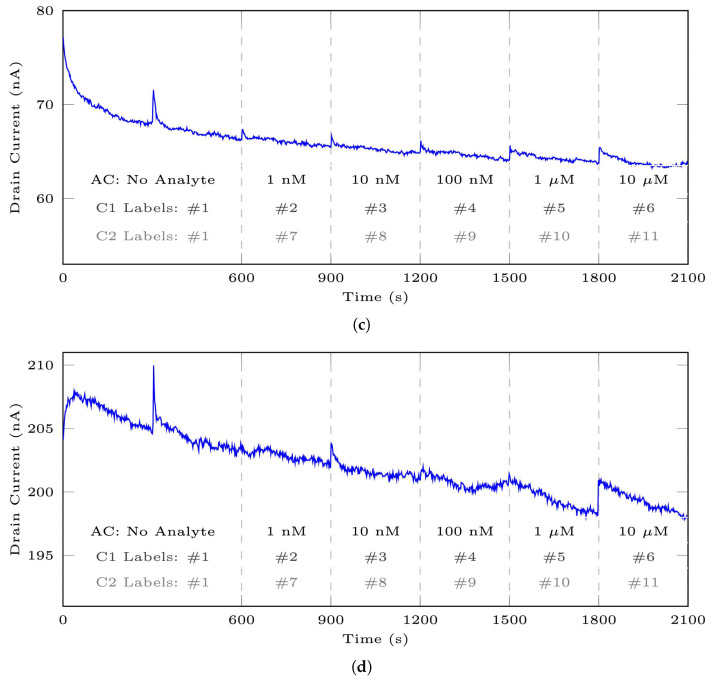
The stereotypical sensing time series registered by aptasensors include (**a**) the typical signal from DS 1 with an initial AC starting from 1 nM, (**b**) another typical signal from the same dataset with AC starting from 1 μM, (**c**) a sample from DS 2, and (**d**) a sample from DS 3. Note that AC, C1 Labels, and C2 Labels refer to the analyte concentration and the segment labels for classification models 1 and 2 in this paper, respectively.

Figure 10 illustrates the stereotypical original signals from the mentioned datasets. Vertical lines are employed to distinguish between various analyte concentrations (ACs) within a signal. It should be noted that the initial ACs for the 35-mer adenosine experiments may not have been the same; the initial AC in Figure 10a was 1 nM and it was 1 μM for Figure 10b. Conversely, the initial ACs for experiments involving the 31-mer and 35-mer oestradiol sensors were entirely the same, as depicted in Figure 10c and Figure 10d, respectively.

Further clarity is needed regarding the terms entire signal and segment, as they are frequently used throughout this paper. In this context, the term entire signal points out all the registered sensing responses from the start to the end of an experiment. Conversely, a segment denotes a specific portion of the entire signal that illustrates the sensor’s registered data to a particular concentration of the target analyte. For example, in Figure 10a, the data at t∈[1,2000] are considered as the entire signal, and this signal consists of three distinct segments: t∈[1,1000] for the No Analyte segment, t∈[1001,1500] for the 1 μM segment, and t∈[1501,2000] for the 10 μM segment.

### 3.2. Data Preparation

There were three steps in order to prepare each dataset for further analysis in this study. These steps include data normalization, segmentation and segment labelling.

#### 3.2.1. Data Normalization

The purpose of feature scaling or data normalization was to standardize the entire signals within a specific dataset to a uniform scale. This process also aimed to prevent certain features from controlling and dominating others. We utilized Z-score scaling for normalization, employing the mean (μ) and standard deviation (σ) calculated from the entire signal.

To demonstrate this concept, take an entire raw signal $X=[x_{1},\ldots ,x_{i},\ldots ,x_{n}]$ of length *n*. Equation (18) outlines the normalization process, producing the normalized signal denoted as $X^{Norm}$, following the method described.

(18) $$X^{Norm}=\left[x_{1}^{Norm},\cdots ,x_{i}^{Norm}=\frac{x_{i}-\mu }{\sigma },\cdots ,x_{n}^{Norm}\right].$$

**Figure 11:**
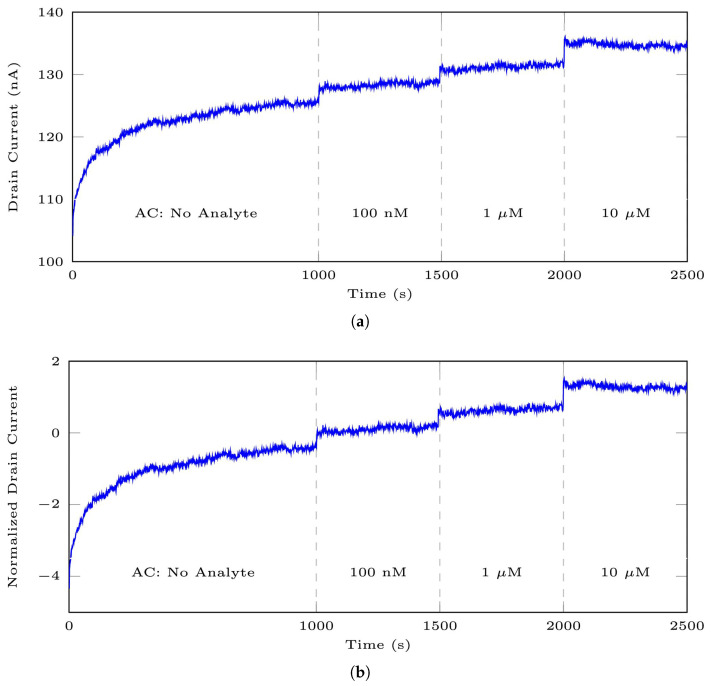
An example of data normalization for the adenosine dataset: (**a**) the raw signal, (**b**) the normalized signal according to Z-score scaling.

Figure 11 displays the effect of Z-score scaling. The impact of the normalization process becomes evident when you compare Figure 11a,b. Figure 11a represents an original signal sample from DS 1, while Figure 11b shows the same signal after undergoing normalization using Equation (18).

It is important to emphasize that the entire signal underwent Z-score scaling. The primary purpose behind normalizing the signals in this manner was to ensure that all signals within each dataset were brought to a consistent scale. Additionally, it should be noted that, for the sake of simplicity, we will subsequently refer to the normalized signals as *X* throughout this paper instead of $X^{Norm}$.

#### 3.2.2. Segmentation

After rescaling the signal, each signal was divided into its component segments. In this study, a segment is defined as a part of a signal with a consistent analyte concentration from beginning to end.

Table 4 displays the total count of segments corresponding to various analyte concentrations in each dataset. It is important to note that the oestradiol signal’s analyte concentration ranged from 1 nM to 10 μM. Consequently, the total count of signals and segments in each class across these datasets remained consistent. On the other hand, the recorded analyte concentration ranges differed among the adenosine signals. Moreover, due to an insufficient number of segments in the 100 pM label in DS 1, this particular class had to be omitted.

Regarding the oestradiol datasets, in the next step, we used the *retime* function in MATLAB R2022b for the resampling to increase the data points from 166 data points in each segment to 300 data points. The reason for resampling was to increase the data points that were essential for the signal extrapolation part.

#### 3.2.3. Anomaly Detection

Anomaly detection is the task of detecting data samples that exhibit statistically distinctive characteristics compared to the other available data instances, regarded as normal instances [32]. Identifying abnormal data is a vital task for machine learning or deep learning scenarios, as abnormal observations hold misinformation and incorrect details and deteriorate the performance of predictive models.

With respect to the signals studied in this research, a number of factors influenced the signals and led to the production of anomalous data. These contributing factors could include complications in the receptor’s immobilization on the surface of CNT, challenges in constructing the sensing interface, transistor malfunctions, difficulties and complications with the receptor on CNT surfaces, laboratory background noise, and so on.

**Figure 12:**
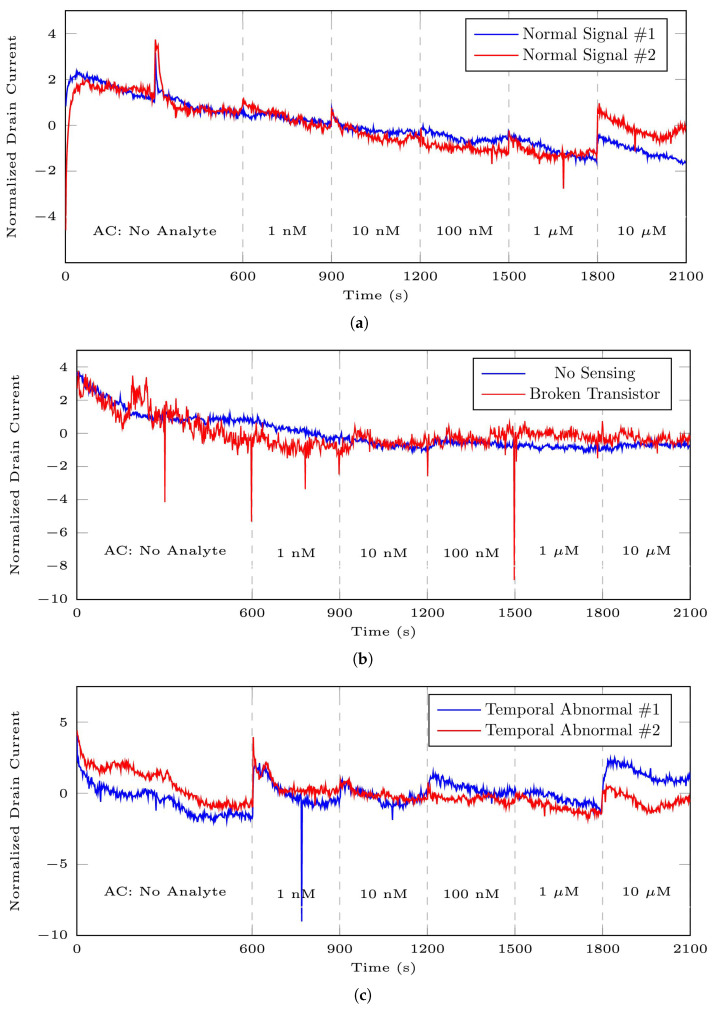
These plots illustrate a comparison between normal 35-mer oestradiol signal patterns and the patterns of abnormal signals: (**a**) signals showing normal and standard behaviour, (**b**) abnormal time series (the blue and red lines represent signals from non-sensing and malfunctioning transistor sensors, respectively), (**c**) signals with abnormal time intervals, captured by functional sensors but exhibiting unusual patterns from 300 to 750 s.

Figure 12 presents a comparison between normal patterns in DS 1 and its abnormal time series. Figure 12a displays the normal pattern. Figure 12b represents two instances of entire signals considered anomalies. The blue and red signals correspond to recordings from non-sensing and broken transistor sensors, respectively. Figure 12c exhibits two signals with abnormal behaviour during a specific time interval, specifically between 300 s and 750 s. In these observations, segments displaying abnormal behaviour were categorized as anomalies, while the others as normal segments. A comprehensive explanation of anomaly description can be found in [16].

The data collector classified the existing segments as standard, borderline, non-sensing signals, and malfunctioning transistors, relying on prior understanding of the sensors’ capabilities and visual data analysis. In our initial study [15], we considered marginal, no-sensing signals and broken transistors as abnormal data or contextual outliers. However, in our subsequent work [16], we introduced an autoencoder-based anomaly detection method as an additional criterion. Consequently, abnormal data in this study were identified using the results of [16] and data visualization. Table 5 displays the total number of normal segments corresponding to each analyte concentration within each dataset after the application of anomaly detection.

#### 3.2.4. Segment Labeling

In this study, as depicted in Figure 1, we developed two distinct classification models. Given the differences between these models and the importance of understanding data classes and labels, we provide an explanation of the segment labelling approach for both models in this section. For a visual overview of the labelling, refer to Figure 10.

Table 6 provides a quick view of the labels in both models C1 and C2. In classification model C1, the objective was to detect and quantify the specific analyte concentration within six different classes, ranging from No Analyte to 10 µM. These models were developed individually for each dataset, excluding abnormal data. In contrast, classification model C2 aimed to distinguish between normal and abnormal segments. For normal segments, model C2 also detected the presence of target analytes and their respective quantities.

It is important to note that all three datasets contain both normal and abnormal data. In the C1 models, abnormal data were excluded, and only normal data were utilized for prediction models. Conversely, in the C2 model, both normal and abnormal data from all three datasets were combined. The approach for generating augmented abnormal data in the C2 model involved creating abnormal data specific to each dataset in the data augmentation process using their respective VAE. Subsequently, the augmented abnormal data from all three datasets were treated as a unified category and labeled as class 12 for the C2 classification model.

### 3.3. Data Preprocessing

The short-time Fourier transform (STFT), introduced by Stockwell [33], is an extension of the Fourier transform that is used for analyzing the time-varying frequency components of non-stationary signals by processing them in short time intervals [34]. This transformation has found application as a data preprocessing technique in signal processing for machine learning and deep learning models [35,36,37,38].

The STFT involves a sliding window to divide a signal into short time segments, often with overlap. Fourier transforms are computed for these short segments, and this process is applied iteratively to cover the entire signal using windowing. In continuous form, the STFT of a single-channel signal x(t) is expressed as Equation (19):

(19) $$STFT_{x(t)}\left(\tau ,\omega \right)=X\left(\tau ,\omega \right)=\int_{-\infty }^{\infty }x\left(t\right)\;\omega \left(t-\tau \right)\;e^{-j\;\omega \tau }\;dt,$$

where ω(τ) represents the analysis window function. It should be noted that in the discrete version of signal *x*(*t*), the integral sign in Equation (19) is replaced by a sum.

The parameters of the window function ω(τ) include the type of window, its length, and the degree of overlap. Various types of window functions are available, such as the rectangular window, Sine window, and Blackman window. Equation (20) describes the calculation of Blackman window:

(20) $$\begin{array}{c} \omega \left[n\right]=a_{0}-a_{1}\;cos\left(\frac{2\pi n}{N}\right)+a_{2}\;cos\left(\frac{4\pi n}{N}\right),\\ a_{0}=\frac{1-\alpha }{2},a_{1}=\frac{1}{2},a_{2}=\frac{\alpha }{2},\\ \alpha =0.16. \end{array}$$

The squared magnitude of the STFT *S* is known as the spectrogram that represents the power spectral density of the function over the joint time-frequency plane, as shown in Equation (21):

(21) $$S_{x(t)}\left(\tau ,\omega \right)={\left|X\left(\tau ,\omega \right)\right|}^{2}.$$

In this work, we used the short-time Fourier transform (STFT) for data preprocessing with a Blackman window. The window parameters were set to a 128-sample Blackman window, with 64 samples of overlap between adjoining segments and a 128-point Fourier transform length.

**Figure 13:**
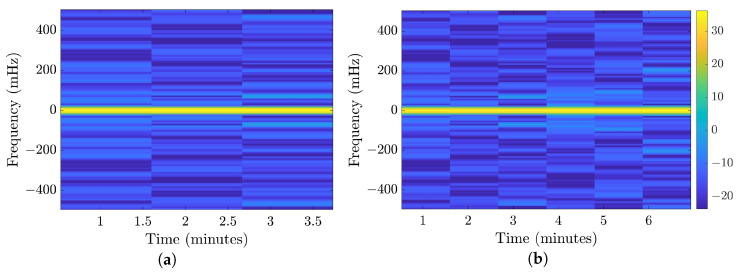
Illustration of the spectrogram for a typical and normal 10 μM 35-mer adenosine segment: (**a**) STFT of the initial 300 s, and (**b**) STFT of the full 500 s.

Figure 13 illustrates the spectrogram of a typical and normal segment from the 35-mer adenosine dataset with an AC of 10 μM. Figure 13a corresponds to the first 300 s of the segment, while Figure 13b shows the spectrogram for the entire 500 s.

The resulting matrices for this transformation, applied to all datasets, were of size 128×3 for the initial 300 s and 128×6 for the full 500 s. These matrices were utilized as the primary input data for all classification models employed in our study. In our analytical assessment, we conducted a comparative evaluation to evaluate the influence of this transformation in contrast to the impact of employing data normalization as a standalone preprocessing technique.

### 3.4. Data Split

Traditionally, data provided for deep learning models is divided into three segments: the training set (60%), the validation set (20%), and the test set (20%) [39]. However, in this study, where we tackled three distinct deep learning tasks—data augmentation, signal extrapolation, and classification—we employed varied strategies for data partitioning.

During the data augmentation phase, 80% of the original datasets were allocated for training, and 20% for validation. The test sets comprised scaled augmented data from [15]. For the signal extrapolation and classification models, we used a split ratio of 60% for training, 20% for validation, and 20% for testing. The data utilized in these stages were the CVAE-augmented data.

## 4. Methods

In this section, we provide a comprehensive overview of the methodologies implemented to tackle the challenges and fulfil the research objectives of our study. Our approach includes the development of three core deep learning models: (1) data augmentation, (2) signal extrapolation, and (3) classification. These models were developed to address the specific requirements of our research tasks. Moreover, it should be noted that the entire implementation process for these deep learning algorithms was conducted using the MATLAB R2022b Deep Learning Toolbox. Moreover, it should be noted that the design of the networks in this study was based on extensive experimentation and evaluation.

### 4.1. Data Augmentation

The exceptional performance of deep learning predictive models relies heavily on the size and consistency of the training datasets to prevent overfitting. However, real-world datasets often suffer from scarcity and class imbalance issues. To effectively increase dataset size and enhance data quality, data augmentation plays a crucial role in the successful application of deep learning models, including time series data [40]. Specifically, generative models like VAE, CVAE, and GAN have been employed for augmenting time series datasets. These models are directly applied to capture the data’s underlying probability distribution, enabling the generation of new samples that closely imitate the original data distribution [41,42].

**Figure 14:**
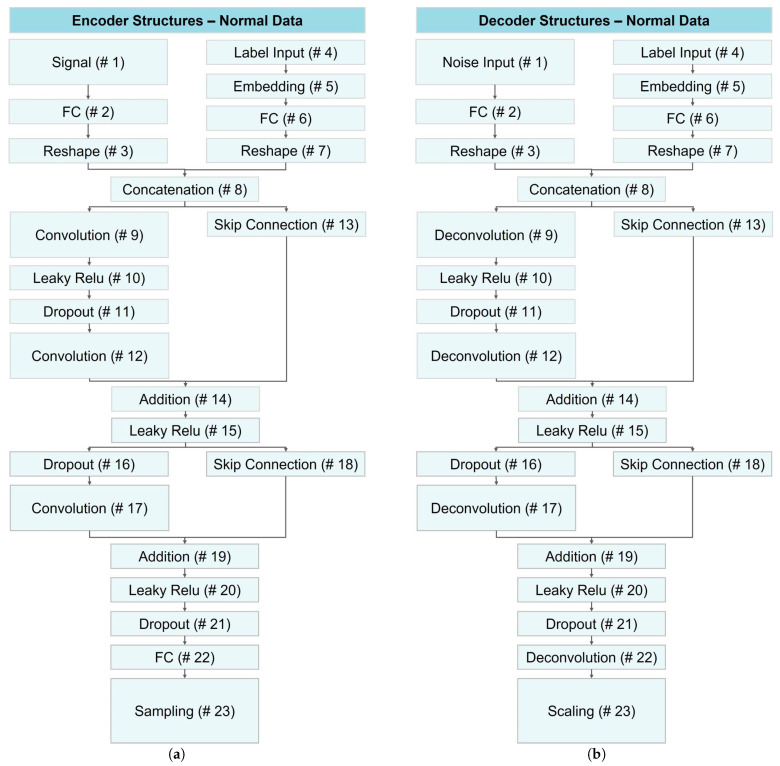
Structure of CVAE networks for data augmentation of normal data: (**a**) the encoder module and (**b**) the decoder network.

In this study, we employed data augmentation using CVAE networks for normal data and VAE for anomaly data to address limited dataset sizes, enabling the development of deep learning classification models with generalization ability and reduced risk of overfitting. Figure 14 illustrates the architecture of encoder and decoder networks utilized for generating normal data across all datasets.

As illustrated in Figure 14, both encoder and decoder networks shared the same architecture with minor variations in layers 1, 9, 12, 22, and 23. In the first layer, the encoder received signal data, while the decoder received random noise. Layers 9 and 12 were convolutional and deconvolutional layers for the encoder and decoder, respectively. In layer 22, the encoder employed a fully connected layer, while the decoder used a deconvolution layer. The last layer differed, with the encoder employing a sampling layer and the decoder using a scaling layer to replicate the original signal.

**Figure 15:**
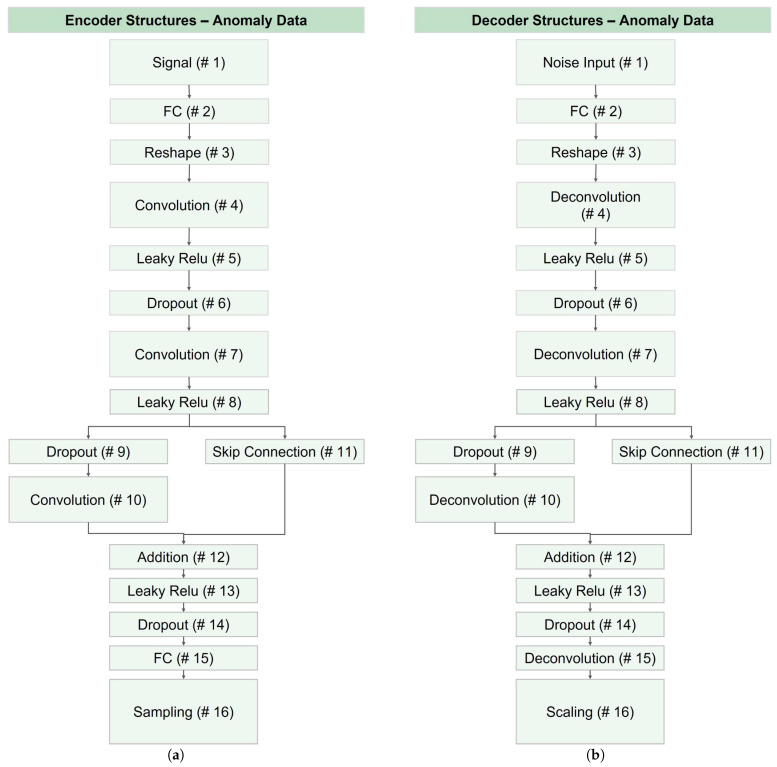
Structure of VAE networks for data augmentation of abnormal data: (**a**) the encoder network, and (**b**) the decoder module. Note that the anomaly data augmentation was essential for C2 models.

Figure 15 illustrates the architecture of encoder and decoder networks utilized for generating abnormal data across all datasets. Notably, AC labels for anomaly data were impractical and lacked meaningful results. Consequently, layers related to label information were omitted from the CVAE, and the corresponding networks were modified to VAE networks. As depicted in this figure, layers 1, 10, 15, and 16 exhibited differences in the encoder and decoder, which was similar to layers differentiation in the CVAE structure.

In the context of the sampling layer, let $X={\left[x_{1},x_{2},\ldots ,x_{m}\right]}^{T}$ denote the input data to this layer, where *m* represents the length of the vector. This vector was a concatenation of the mean vector $\mu_{z}$ and the variance vector $\Sigma_{z}$, namely, $\mu_{z}={\left[x_{1},\ldots ,x_{m/2}\right]}^{T}$ and $\Sigma_{z}={\left[x_{(m/2)+1},\ldots ,x_{m}\right]}^{T}$. The sampling layer produced three outputs: $\mu_{z}$, $\sigma_{z}$, and *Z*. Equations (22) and (23) describe the calculations for these outputs:

(22) $$\sigma_{z}=e^{\frac{1}{2}\;\Sigma_{z}},$$

(23) $$Z=\epsilon \cdot \sigma_{z}+\mu_{z},$$

where ϵ was a vector with normally distributed random numbers generated with *randn* function in MATLAB R2022b.

Regarding the scaling layer, Equation (24) represents the function utilized in this layer to scale and map the input vector *X* to the output data $f_{s}\left(X\right)$ while maintaining the same scale as the original registered data:

(24) $$f_{s}\left(X\right)=\alpha \cdot tanh\left(X\right),$$

where α∈R was the variable α that varied according to the dataset scale. For example, for normal data across all datasets, we set α to 1.5, which aligns with the normalized drain current segments for normal data, lying within the range of [−1.5,1.5]. Conversely, when generating anomalies in the 35-mer adenosine dataset, α is set to 5, reflecting the approximate range of abnormal data within [−5,5].

### 4.2. Signal Extrapolation

Time series forecasting using deep learning has gained prominence in academic research across diverse domains [43,44]. In the sensors industry, deep learning-based time series modelling has found application in tasks such as denoising, dimensionality reduction, anomaly detection, structural damage identification, and predicting future sensor outputs [45]. Various deep learning algorithms have been employed for forecasting time series data, including CNN, temporal convolutional network TCN, LSTM, and GRU [43,44,45].

In this section, we employed recurrent-based networks for forecasting future sensor outputs in the oestradiol datasets, a task we simply refer to as signal extrapolation in this study. Our motivation for this approach stemmed from the results of our previous work [15,16], where we raised concerns about the impact of signal length on classification and anomaly detection outcomes. In both of those studies, deep learning models performed better when using the 35-mer adenosine dataset with segments lasting 500 s, as opposed to the 31-mer and 35-mer oestradiol datasets with segments lasting 300 s. As a result, we applied recurrent-based networks to extrapolate the oestradiol datasets from 300 s to 500 s.

**Figure 16:**
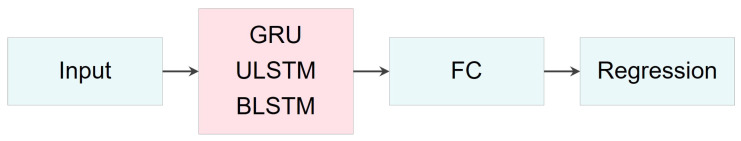
A schematic of the network architecture. The primary distinction among these networks lay in their recurrent layer, which incorporated either a GRU, unidirectional LSTM, or bidirectional LSTM layer.

Figure 16 visualizes the network architecture used for signal extrapolation. These networks comprised four successive layers: a sequential input layer, a recurrent layer, a fully connected layer, and a regression layer. The primary distinction among these networks lay in their recurrent layer, which incorporated either a GRU, unidirectional LSTM, or bidirectional LSTM layer. For a more detailed comparison of the network layers and parameters, please refer to Table 7.

Remember that the input layer’s size fed into the networks matched the length of the segments and was treated as a single sequence. Similarly, the output size of the networks was a sequence with a length identical to that of the input segment. Also, the *n* refers to the number of hidden units in the recurrent layers. In this section, the number of hidden units, *n*, was set to 128. Note that the input weights, along with the recurrent weight and bias matrices, were combined to create the input weights for both GRU and LSTMs ($W_{x}=\left[W_{rx};W_{zx};W_{\tilde{h}x}\right]$, $W_{x}=\left[W_{ix};W_{fx};W_{cx};W_{ox}\right]$), recurrent weights in the same order ($W_{h}=\left[W_{rh};W_{zh};W_{\tilde{h}h}\right]$, $W_{h}=\left[W_{ih};W_{fh};W_{ch};W_{oh}\right]$), and bias in the same order ($b=[b_{r};b_{z};b_{\tilde{h}}]$, $b=[b_{i};b_{f};b_{c};b_{o}]$).

The pseudocode outlined in Algorithm 1 describes the technique used to extend the length of data segments to meet a specified desired length, set at 500 s for this study. With an input dataset denoted as *D* and an initial data segment, *X*, the algorithm proceeded by iteratively predicting future sensor readings at variable time intervals, controlled by the timeStep parameter, and appending these predictions to the existing data. This process continued until the desired data segment length was achieved. Key steps included window-based data preparation, neural network training, and iterative prediction updates.

**Figure 17:**
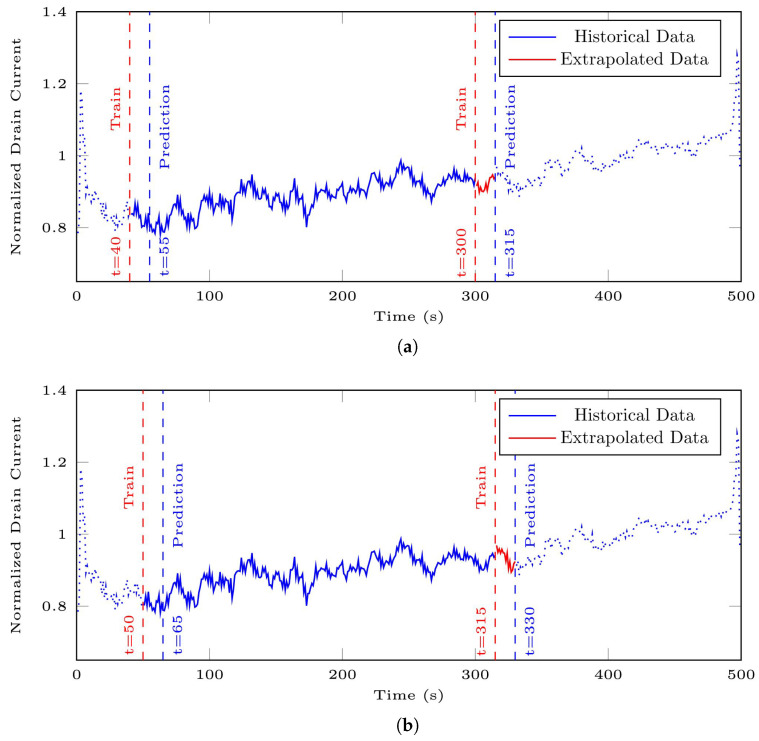
Illustration of two iterations of signal extrapolation procedure on a segment of the 35-mer adenosine dataset with AC of 10 μM: (**a**) first iteration, and (**b**) second iteration.

Figure 17 illustrates the application of the algorithms described on a segment with AC of 10 μM from the 35-mer adenosine dataset. In the first iteration, as visualized in Figure 17a, the predictor window covered the time interval [40,300], which was used to predict the subsequent time interval [55,315]. Consequently, the data from the interval [301,315], as the extrapolated part, was appended to the actual segment.

During the second iteration, according to Figure 17b, the predictor and predicted segments were adjusted to [50,315] and [65,330], respectively. As a result, the data from the interval [316,330], as the extrapolated part, was appended to the actual segment.
**Algorithm 1** Algorithm for forecasting future sensor outputs**Input**: Dataset *D*, Segment $X=[x_{1},\ldots ,x_{n}]$, Desired Length *L***Output**: Extrapolated Segment $X_{e}=\left[x_{1},\ldots ,x_{n},x_{n+1},\ldots ,x_{L}\right]$$X_{e}\leftarrow X$l←SegmentLengthstartPoint←StartPointtimeStep←15stepOffset←0**while** 
l≤L **do**   **for all** X∈D **do**         predictorStart←startPoint+stepOffset         predictorEnd←l−timeStep         $\mathrm{Predictor}\leftarrow [x_{\mathrm{predictorStart}},\ldots ,x_{\mathrm{predictorEnd}}]$         targetStart←predictorStart+timeStep+stepOffset         targetEnd←l         $\mathrm{Target}\leftarrow [x_{\mathrm{targetStart}},\ldots ,x_{\mathrm{targetEnd}}]$   **end for**   TrainNetwork(Predictor,Target)   $\mathrm{PredictFutureDataPoints}\;X_{p}=\left[x_{l+1},\ldots ,x_{l+\mathrm{timeStep}}\right]$   $X_{e}\leftarrow \mathrm{Concatenate}\left(X_{e},X_{p}\right)$   $l\leftarrow \mathrm{Length}(X_{e})$   stepOffset←stepOffset+10**end while**

### 4.3. Classification Models Architectures

As explained before, in this study, we have developed two distinct classification models: C1 and C2 models. The primary objective of the C1 models is to detect and quantify analyte concentrations for each dataset individually. On the other hand, the C2 model is designed to identify abnormal and normal data, detect various analytes, and quantify their respective concentrations throughout all available datasets.

Seven distinct deep learning models were employed for the classification tasks: (1) GRU, (2) ULSTM, (3) BLSTM, (4) ConvGRU, (5) ConvULSTM, (6) ConvBLSTM, (7) CNN. The architectures of these networks remained consistent across both the C1 and C2 models. The only difference in the network architectures was in their classification output layers: the C1 networks had output layers with a size of six classes, whereas the C2 models’ output layers consisted of twelve classes.

**Figure 18:**
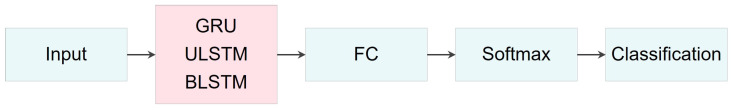
A schematic of the recurrent-based network architecture for classification models. The primary distinction among these networks lay in their second layer, which included either a GRU, ULSTM, or BLSTM layer.

Figure 18 and Table 8 visualize and describe the three employed RNN-based networks for both classification models and compare the networks with regard to their parameters in different layers. These networks consisted of 5 consecutive layers: (1) a sequential input layer, (2) a recurrent-based, (3) a fully connected layer, (4) a Softmax layer, and (5) a classification layer. The main differences among these networks were in their second layers, with the implementation of GRU, ULSTM, and BLSTM structures within the recurrent layer.

Note that *n* and *m* refer to the hidden node numbers in the recurrent layer and the class numbers in the classification models, respectively. In this part, the hidden node numbers, *n*, was set to 128, and *m* for C1 and C2 models were 6 and 12, respectively. Furthermore, the details of the input weights ($W_{x}$), recurrent weights ($W_{h}$), and biases (*b*) have been previously explained in this section.

**Figure 19:**
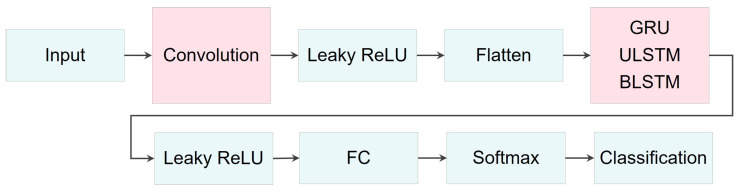
Illustration of ConvRNN networks architectures used for enhanced feature extraction in classification tasks.

Moreover, Figure 19 illustrates the three proposed networks for classification, while Table 9 provides detailed descriptions and relevant information about their architectures, collectively referred to as ConvRNNs. These networks, namely ConvGRU, ConvULSTM, and ConvBLSTM, differ from the previously mentioned RNN-based networks by incorporating a 2D convolutional layer after the input layer. Some structural modifications were made to refine and fine-tune the networks.

Note that the variable $l_{s}$ represents the length of the segments that are fed into the networks. In our earlier models, the input layers were configured as sequential input layers. However, in the case of the ConvRNNs, we opted for an image input layer. As a result, the input size for the sequential layers was set to one, while for the image input layer, it matched the length of the segments.

The variable $l_{c}$, as the output size of the convolution layers, was calculated according to Equation (13). Additionally, $s_{f}$ and $n_{f}$ denote the size and number of filters in the 2D convolutional layer, respectively, with *p* representing padding and *s* indicating the stride size for this layer. In these classification models, the mentioned variables were set as follows: $l_{s}\in \left\{300,500\right\}$, $s_{f}=5$, $n_{f}=32$, p=1, s=1, n=128, and m∈{6,12}.

It is important to note that the variable $l_{s}$ represents the input length of the segments fed into the networks, which can vary between 300 s and 500 s. This variation allowed us to assess the impact of segment length on the models’ performance.

**Figure 20:**
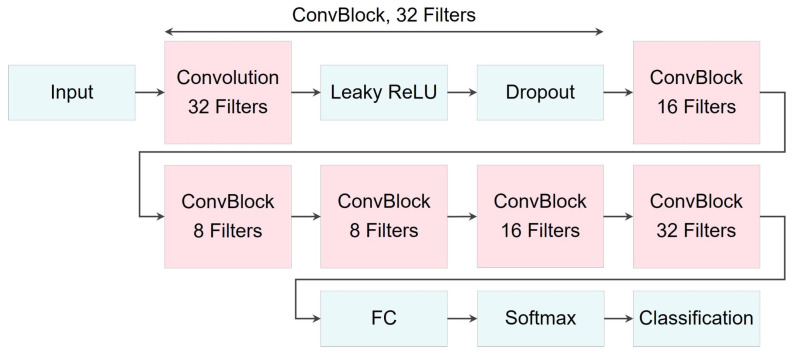
Illustration of CNN network architecture used for classification models.

Finally, Figure 20 provides an illustration of the proposed CNN architecture used for classification, while Table 10 offers a detailed description of its layers.

The variables $l_{c_{i}}$ for i=1,…,6, as the output size of the convolution layers, were calculated according to Equation (13), where filters’ size $s_{f}=5$, padding p=1, and stride s=1. Also, the number of filters were set as follows: $n_{f_{1}}=32$, $n_{f_{2}}=16$, and $n_{f_{3}}=8$.

### 4.4. Model Performance Evaluation

There are two approaches for the evaluation of deep learning models in this study, which are prediction and classification.

#### 4.4.1. Prediction Model Evaluation Metrics

Regarding the evaluation of the data augmentation and signal extrapolation methods, we reconstructed their corresponding test data using the training networks and then calculated the reconstruction error. This error quantifies the dissimilarity between the original data and the reconstructed output and can be measured using statistical metrics such as mean absolute error (MAE) [46] or mean square error (MSE) [47]. In this study, we employed MSE as the metric for calculating the reconstruction error.

Consider *X* as an input segment represented by $\left[x_{1},x_{2},\ldots ,x_{N}\right]$, and let $\hat{X}$ represent its reconstructed output given by $\left[{\hat{x}}_{1},{\hat{x}}_{2},\ldots ,{\hat{x}}_{N}\right]$. Equation (25) defines MSE as the metric for measuring reconstruction errors:

(25) $$\mathrm{MSE}=\frac{1}{N}\sum_{i=1}^{n}{\left({\hat{x}}_{i}-x_{i}\right)}^{2}.$$

#### 4.4.2. Classification Model Evaluation Metrics

The classification performance of C1 and C2 models was assessed by the overall accuracy (ACC) and the Macro F1-score (MF1). To calculate these metrics, initially, a confusion matrix was created using the classification of the test data, and subsequently, the two mentioned metrics were calculated.

The overall accuracy, as described in Equation (26), represents the proportion of truly classified elements in the test data out of the total elements. It was calculated by adding the diagonal elements of the confusion matrix and dividing the sum by the total number of elements.

(26) $$\mathrm{ACC}=\frac{\mathrm{Number}\;\mathrm{of}\;\mathrm{correct}\;\mathrm{preditions}}{\mathrm{The}\;\mathrm{overall}\;\mathrm{count}\;\mathrm{of}\;\mathrm{items}\;\mathrm{in}\;\mathrm{the}\;\mathrm{test}\;\mathrm{set}}.$$

The Macro F1-score serves as a valuable metric for classification models dealing with multiple classes. To comprehend how the macro F1-score is computed, it is crucial to understand its constituent elements: recall, precision, and F1-score. Precision in Equation (27), also known as positive predicted value (PPV), represents the ratio of accurately classified positive instances out of all the instances predicted positive by the model. Recall in Equation (28), denoted as the true positive rate (TPR), signifies the proportion of accurately classified positive instances out of all actual positive instances. The F1-score, outlined in Equation (29), is calculated from precision and recall. Finally, the macro F1-score, described in Equation (30), calculates the average of class-wise F1-scores concerning the model.

(27) $$\mathrm{Precision}=\mathrm{PPV}=\frac{\mathrm{TP}}{\mathrm{TP}\;+\;\mathrm{FP}},$$

(28) $$\mathrm{Recall}=\mathrm{TPR}=\frac{\mathrm{TP}\;}{\mathrm{TP}\;+\;\mathrm{FN}},$$

(29) $$F1\text{-}\mathrm{score}=\frac{2}{1/\mathrm{precision}+1/\mathrm{recall}},$$

(30) $$\mathrm{Macro}\;F1\text{-}\mathrm{score}=\frac{1}{m}\sum_{i=1}^{m}{\left\{F1\text{-}\mathrm{score}\right\}}_{i},$$

In these equations, *m* represents the total number of classes within a specific classification model.

## 5. Results

### 5.1. Data Augmentation

Table 11 presents detailed information about the architecture of the networks and the layers’ output sizes that were utilized for data augmentation. These networks are comprised of both encoder and decoder modules [10]. The CVAE networks were employed to augment normal data, primarily due to variations in AC labels across available datasets. In contrast, the VAE networks were used for generating anomaly data, as these signals lacked different labels, making the use of AC labels impractical. Additionally, since the sizes of the segments related to the 31-mer and 35-mer oestradiol datasets, referred to as DS 2 and DS 3 were similar, their network information was merged in one column. In contrast, the information related to 35-mer adenosine as DS 1 remained separate.

Table 12 shows two types of information regarding the data augmentation with CVAE for normal data on the three available datasets: (1) the performance evaluation of the networks and (2) examples of augmented normal segments. After training the networks as described, we initially evaluated their performance using segments generated with a scaling data augmentation method, as previously detailed in our work [15]. These generated segments served as the test data, since the original datasets lacked sufficient data for testing purposes. Therefore, we first reconstructed the test data using the trained CVAE networks. The examples of reconstructed normal segments are shown in the left column of Table 12, while the middle column presents the histogram of reconstruction errors calculated using mean square error (MSE) according to [48].

Subsequently, the decoder modules of the networks were employed to generate new data. For each AC, the corresponding label was input into the feature input layer, and the desired amount of data was augmented. The examples of augmented data are visualized in the right column of Table 12. The total size of both the original and augmented data for each AC was set to 200. Likewise, the anomaly data was generated with VAEs’ decoder modules.

### 5.2. Signal Extrapolation

In the signal extrapolation phase, we employed the 35-mer adenosine dataset for forecasting future sensor outputs. This dataset had segments with a length of 500 s, which was longer than the segments in both oestradiol datasets, each with a length of 300 s. This longer segment length provided a suitable basis for evaluating the performance of the recurrent-based prediction models in signal extrapolation. Specifically, we used the initial 300 s as the predictor and extrapolated the subsequent 200 s.

The data used here for training and then evaluating the models were the original and augmented data with CVAE, explained in the previous section. The MSE was used as the statistical metric to calculate the prediction error, and to assess and compare the performance of the prediction models. In this part, MSE calculated the difference between the original segments and their corresponding extrapolated parts at the final 200 s.

**Figure 21:**
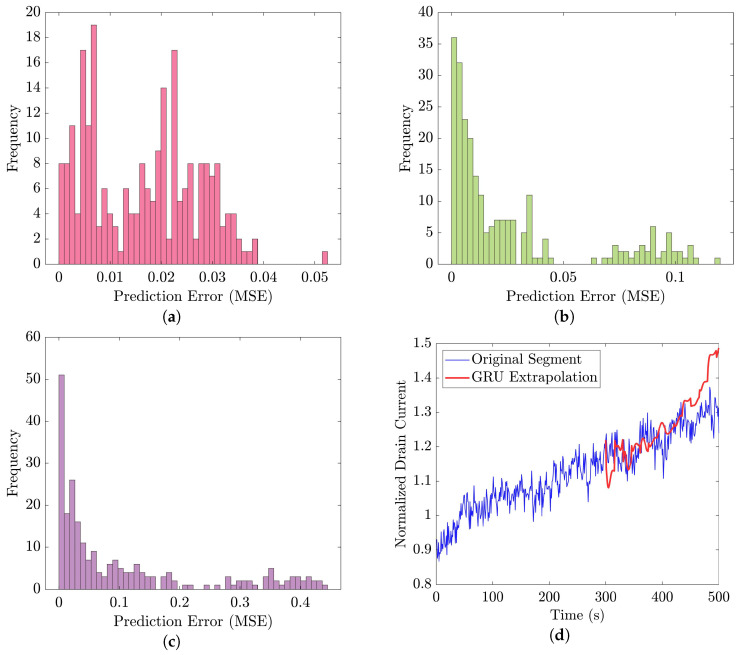
Performance evaluation and an example of signal extrapolation on the 35-mer adenosine dataset: histograms of prediction error for (**a**) GRU, (**b**) ULSTM, and (**c**) BLSTM networks, along with (**d**) an example of forecasting future sensor outputs with three GRU-based prediction models at AC of 10 μM.

Figure 21 displays the results of the signal extrapolation process. Figure 21a–c show histograms of the prediction error assessed by MSE for each prediction model. Additionally, Figure 21d provides an example of extrapolated outputs using the GRU network.

**Figure 22:**
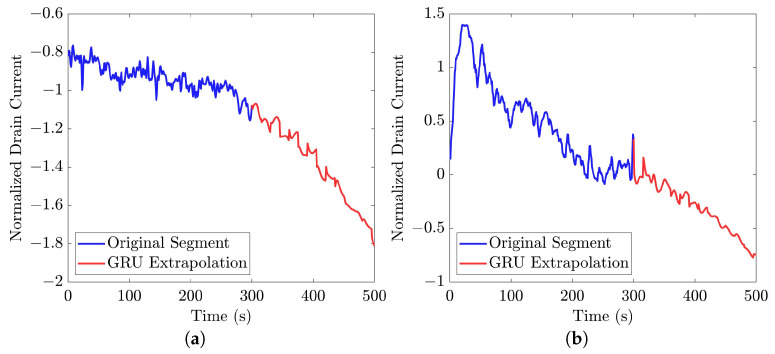
Examples of signal extrapolation with GRU network on oestradiol datasets: (**a**) a 31-mer Oestradiol segment with AC of 1 μM, (**b**) a 35-mer Oestradiol segment with AC of 100 nM.

Similar to [13], as shown in these histograms, we observed that the GRU network outperformed the other two networks. Furthermore, as indicated in Table 7, the GRU model had fewer learnable parameters compared to ULSTM and BLSTM, resulting in shorter training times for computation than the LSTM models. Thus, we implemented the signal extrapolation procedure on the oestradiol datasets with a GRU-based prediction model. Subsequently, Figure 22 represents examples of the predicted outputs from the oestradiol datasets.

### 5.3. Classification Models

In this section, we delve into the comprehensive performance evaluation of the C1 and C2 models on their respective test sets. Our study focused on three key aspects: data preprocessing methods, signal extension techniques, and the comparative analysis of various deep learning-based neural networks for classification.

Regarding the data preprocessing, we compared the impact of two approaches: normalized data with z-score scaling and normalized data with the STFT method. For signal extrapolation, we examined the effect of segment lengths, specifically 300 s and 500 s, on the classification outcomes. Finally, our study culminated in a thorough comparison across these four categories, employing the explained deep learning algorithms.

The recurrent layers in the algorithms were configured with 128 hidden units when applicable. According to our previous work [15], the number of hidden units did not greatly affect the networks’s performance. Thus, in this study, we just considered one number, 128. The training process comprised 100 epochs with a minibatch size of 128, utilizing the Adam optimization algorithm. The learning rate was set to 0.002. Moreover, to enhance the robustness of the prediction models and avoid the risk of overfitting, K-fold cross-validation with k = 10 was applied. All other hyperparameters were maintained at their default values in MATLAB R2022b.

#### 5.3.1. C1 Models

**Figure 23:**
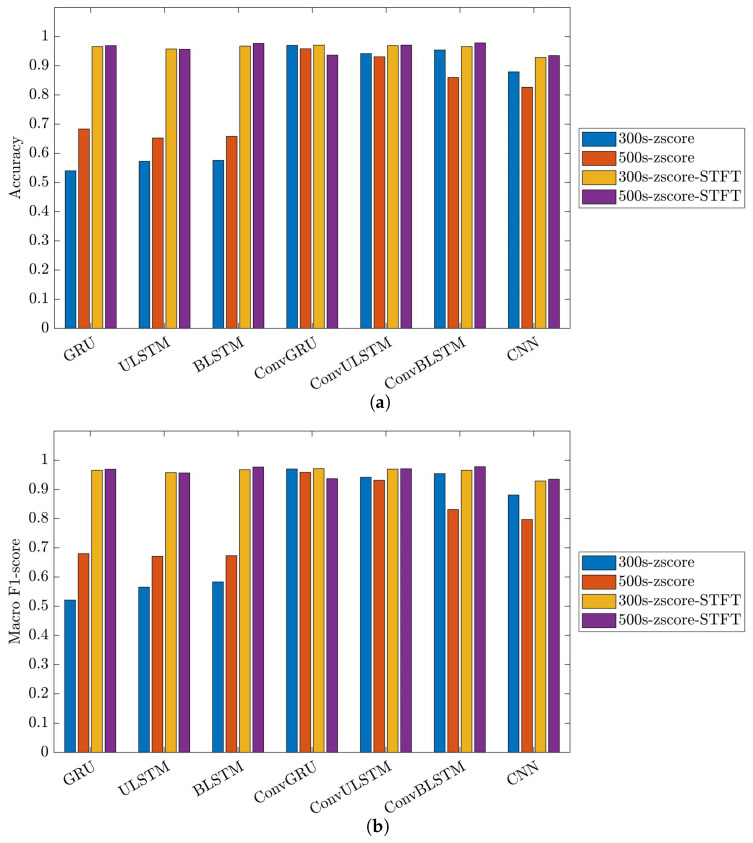
Performance metrics of C1 model on 35-mer adenosine dataset: (**a**) accuracy and (**b**) macro F1-score.

Figure 23 illustrates the performance and impact of deep learning models on the 35-mer adenosine dataset. It is important to note that in C1 models, there were six distinct classes. This configuration implies that the primary objective of the classification model was to individually identify and quantify the analyte concentration for each dataset.

**Figure 24:**
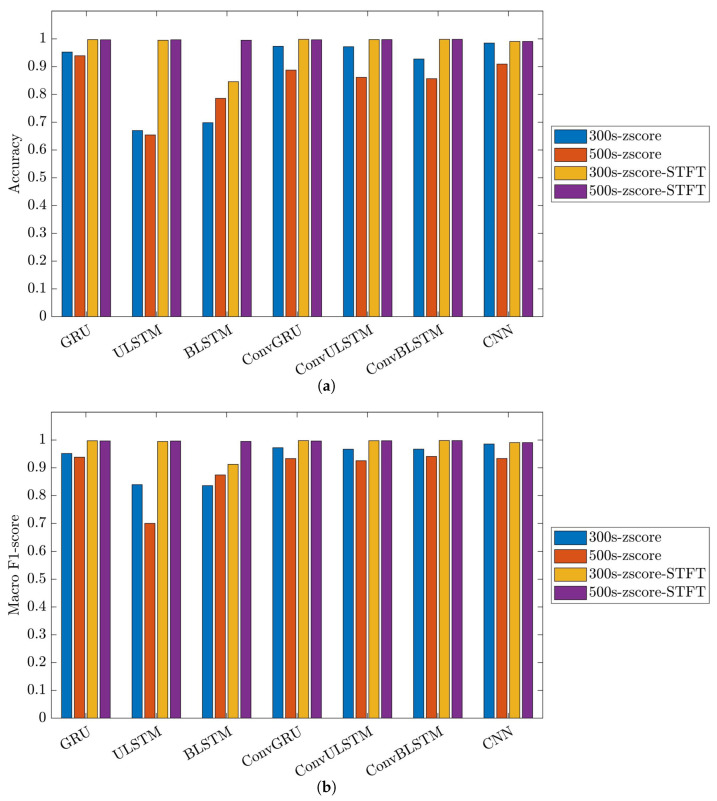
Performance metrics of C1 model on 31-mer oestradiol dataset: (**a**) accuracy and (**b**) macro F1-score.

**Figure 25:**
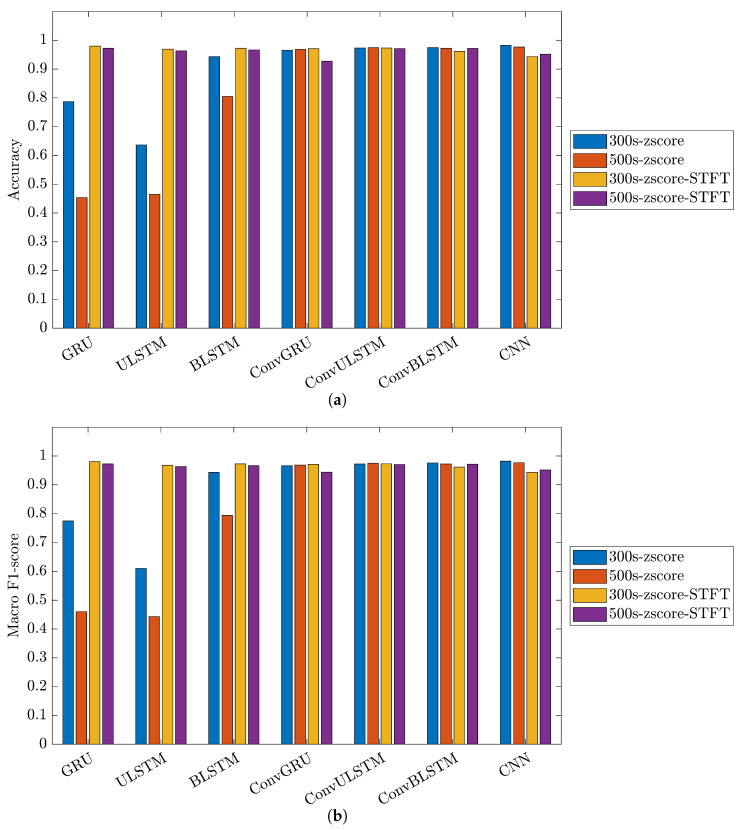
Performance metrics of C1 model on 35-mer oestradiol dataset: (**a**) accuracy and (**b**) macro F1-score.

Figure 24 and Figure 25 showcase the performance and impact of deep learning models on the 31-mer and 35-mer oestradiol datasets, respectively.

The choice of the data processing method significantly influenced the classification outcomes. Utilizing the STFT method as the data preprocessing technique had a noticeable effect on the employed classification models. As a result, all the networks for the three datasets yielded comparable, consistent and high-performance results, ranging between 84% to 99% in both metrics, i.e., accuracy and macro F1-score.

Comparing the deep learning networks, it is evident that ConvGRU, ConvULSTM, ConvBLSTM, and CNN models demonstrated consistent and comparable performance, surpassing RNNs across all datasets.

In the context of segment length, it can be deduced that segment length played a crucial role in recurrent networks such as GRU, ULSTM, and BLSTM models just in the 35-mer adenosine dataset. In the case of the 35-mer adenosine dataset, using the lengthier segments resulted in a notable accuracy boost, reaching up to 8% in the ULSTM and BLSTM models and as high as 15% in the GRU model. However, it did not affect networks with convolution layers in their structures.

Considering signal extension for the oestradiol datasets, it is important to note that this method did not prove effective in our developed classification models. Nevertheless, its inclusion in this study does not support our previous hypothesis regarding the significance of segment length for these two datasets in predictive modelling [15,16].

#### 5.3.2. C2 Model

**Figure 26:**
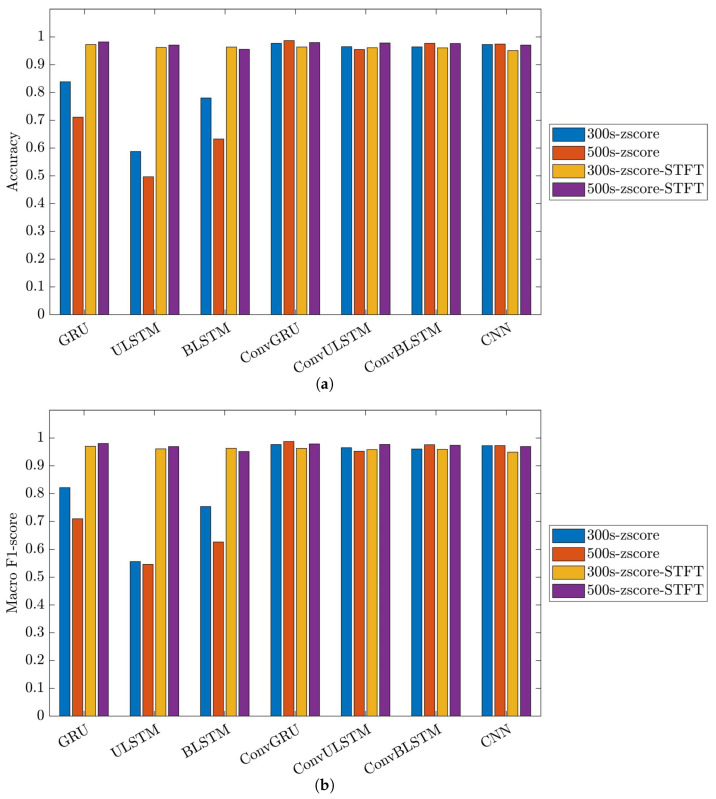
Performance metrics of C2 model: (**a**) accuracy and (**b**) macro F1-score.

Figure 26 showcases the performance and impact of deep learning models on the C2 classification model.

The outcomes of this model closely mirrored those of the C1 models. It is evident that employing STFT as the data preprocessing method significantly enhanced the performance of classification tasks across all networks. The accuracy rates for networks utilizing STFT ranged between approximately 95% and 98%, underlining its consistent effectiveness in improving classification outcomes.

In the context of signal extrapolation, it is crucial to note that this method did not demonstrate effectiveness in our developed classification. Specifically, the results for GRU and ULSTM models using extrapolated segments were consistently lower than those with original segments, showing a decrease of at least 10%.

Upon comparing the networks, it can be deduced that models with convolution layers consistently delivered stable results, outperforming RNNs across various signal lengths and data preprocessing techniques, showcasing high performance.

## 6. Discussion

Aptasensors utilizing nanomaterials are valuable biosensors as they can detect minute chemicals and species. A key objective in biosensor development is identifying and measuring trace amounts of specific analytes. Deep learning techniques have become highly appealing tools for advancing biosensors and their data analysis.

Deep learning generative algorithms, like GANs or VAEs, provide a valuable solution for data augmentation, addressing the limitations of original data collection. They offer a cost-effective and time-saving alternative for analysis, crucial in biosensor development [49]. Additionally, deep learning algorithms have been extensively utilized in time series forecasting, allowing the accurate prediction of future data points [12,50]. This forecasting approach is effective in tasks such as sensor signal extrapolation and predicting future outputs. Moreover, deep learning networks serve as powerful tools for various tasks, including identification, quantification, and classification models [51,52]. These methods utilize advanced neural network structures to handle intricate data patterns, making them indispensable in both contemporary research and industrial applications.

In this work, we have successfully proposed and compared several classification models designed to automatically identify and measure specific analytes based on segments of signals captured by the aptasensors. However, addressing the challenge of inadequate dataset sizes, common in real-world scenarios, remains crucial to achieving our primary goals. Additionally, employing a signal extrapolation method was essential to ensure the uniformity and equality of all available datasets’ lengths. This step was necessary to test our earlier hypothesis, which was proposed in our previous papers and involved variations in segment lengths [15,16].

Regarding the data augmentation, we designed and employed CVAE and VAE networks to augment the normal and abnormal data, respectively. The data augmentation and network designs were applied separately for each dataset. The networks were trained by all available and original segments within their corresponding datasets. In the test phase, we used the data that were generated by the proposed scaling data augmentation in [15] as the test sets. Finally, MSE was used as the evaluation metric for calculating the reconstruction error. The potential contribution of the presented data augmentation method is that it can be reused for anomaly detection or outlier detection.

In the context of signal extrapolation, we designed and compared networks incorporating recurrent layers to generate segments with uniform lengths [53]. For training and testing the signal extrapolation algorithm, we utilized the 35-mer adenosine dataset, which featured 500-s data points and was longer than the two oestradiol datasets. This dataset allowed us to thoroughly assess the proposed algorithm. To evaluate prediction accuracy, MSE was employed, comparing real data with extrapolated data in the last 200 s. Among the three recurrent networks studied, the GRU-based network outperformed the other two LSTM-based networks [13]. Subsequently, this technique was applied to the oestradiol datasets to extend the segments from 300 s to 500 s. Furthermore, this proposed signal extrapolation can be regarded as an algorithm for time series forecasting for similar datasets.

We categorized our classification objectives into two models: C1 and C2. C1 models were tailored for individual datasets, focusing on six classes that spanned from zero analyte presence to 10 μM concentrations. Conversely, the C2 model considered all datasets, distinguishing 12 classes. These classifications included identifying normal and abnormal data, detecting analyte presence, and measuring its concentration when present.

We designed seven deep learning networks incorporating recurrent, convolutional, or hybrid layers to achieve our classification objectives. These networks were subjected to two preprocessing methods: z-score scaling and z-score combined with the STFT method. Furthermore, we examined the influence of segment length on the classification outcomes. Evaluation metrics included overall accuracy and macro F1-score, given the presence of multiple classes within each classification model.

The results indicated a significant improvement in model performance, particularly in recurrent-based networks, when utilizing the STFT method as a data preprocessing technique. However, the signal extrapolation method did not yield consistent effects on classification models. Networks combining both recurrent and convolutional layers consistently outperformed others. Interestingly, the performance of CNN networks was comparable to that of Conv-RNN networks [38].

In our analysis, we observed that the C1 models and RNN networks achieved lower accuracy levels with the 35-mer oestradiol dataset, consistent with the findings in [15]. However, when accounting for randomness, all datasets exhibited similar results in other networks.

In our future research, we plan to explore deep transfer learning techniques such as VGG-19, Inception, and ResNet-50 for our classification models. This will enable us to assess and compare their impact on the overall classification performance [54,55]. Additionally, we intend to incorporate attention mechanisms into our classification models. Attention mechanisms can improve feature extraction from input data, potentially enhancing the overall performance of our models [56]. We also can use hybrid networks [57] or CNN networks [58] for signal extrapolation to examine and compare their effects on time series forecasting.

The limitation in our study came from not having sufficient data. The first limitation was related to the signal analysis technique used. In machine learning tasks, it is typical to apply the same changes to both training and test data. However, we employed a method to scale each time series derived from its mean and standard deviation. This occurred due to the unfeasibility of calculating the mean and standard deviation using the existing statistical techniques. The second limitation was related to the lack of data for testing the CVAE networks. Thus, we used the scaling augmented data from our previous work. The third limitation was related to the data augmentation with VAE for anomaly data. There was insufficient abnormal data to evaluate the VAE networks for augmented data.

## 7. Conclusions

In this paper, we proposed three main techniques utilizing deep learning algorithms to examine the signals from the drain current of three comparable electrochemical sensors across the datasets named the 35-mer adenosine, and the 31-mer and 35-mer oestradiol. These signals captured the sensors’ responses as the concentrations of the target substances gradually rose from 1 nM to 10 μM. Our primary goal was to achieve automatic identification and measurement of specific analytes based on a section of the signal captured by these sensors. These three main steps to fulfil our goal can be categorized into (1) data augmentation, (2) signal extrapolation, and (3) classification models.

The CVAE-based data augmentation method proved highly effective in enhancing and strengthening the generalization abilities of classification models, while only insufficient original data was provided. Furthermore, the results indicate that this augmentation technique was particularly impactful when coupled with the STFT method as the data preprocessing technique.

We used a signal extrapolation method to generate uniform signal lengths for all the available datasets as the feed for classification models. The proposed GRU-based signal extrapolation algorithm demonstrated exceptional performance in reconstructing original data within the 35-mer adenosine dataset, which featured lengthier signals. Consequently, this algorithm was applied to extrapolate oestradiol datasets with segments lasting 300 s. Notably, while this method did not yield effective results in our classification models, its inclusion in the study was pivotal for investigating our hypothesis concerning the significance of segment length in predictive modelling. Ultimately, the findings of this study do not support our initial hypothesis.

In terms of classification models, initially, we designed seven deep learning networks with the aim of detecting and quantifying specific analyte concentrations across six distinct classes. These classes ranged from the absence of the analyte to 10 μM, with each dataset’s segments being analyzed independently. The classification performance was evaluated with two metrics: accuracy and macro F1-score. The outcomes indicated that ConvGRU, ConvULSTM, ConvBLSTM, and CNN exhibited robustness and consistently delivered high-performance results compared to recurrent networks.

In the second classification model, we employed the same set of deep learning networks for a twelve-class classification task. The objective was to differentiate abnormal data from normal segments, ascertain the absence or presence of the analytes in the sample, and, if an analyte was present, identify the specific substance and quantify its concentration. The results mirrored those of the previous model, demonstrating that ConvGRU, ConvULSTM, ConvBLSTM, and CNN exhibited stability and consistently outperformed recurrent networks.

The outstanding performance of ConvGRU, ConvULSTM, ConvBLSTM, and CNN suggests that sequential data analysis does not necessarily yield better results with recurrent networks, which are specifically designed for such data. Our findings indicate that the combination of convolutional and recurrent layers, as seen in ConvRNN networks, outperforms pure RNN architectures, even with the increase in learnable parameters. This suggests that, in the context of our datasets, the optimal classification strategy involves using both recurrent and convolutional layers for effective time series feature extraction.

Additionally, our results highlight the efficiency of CNN models, which possess fewer learnable parameters yet demonstrate computational efficiency and speed and satisfactory classification performance when compared to ConvRNN models. This underscores the versatility of CNNs in handling time series data with remarkable efficiency and accuracy. The evaluation metrics of the classification models indicate that the proposed networks are effective tools for automatic identification and measurement of analyte concentration.

Furthermore, our results indicate that the exceptional performance of these networks can be effectively enhanced by employing an appropriate data preprocessing technique, such as the STFT method, as a time-frequency feature extraction utilized in our study. This suggests that the choice of data preprocessing technique could serve as a beneficial tool for improving neural network performance, aligning with our goal of automatic identification and measurement of specific analytes.

## Figures and Tables

**Table 1:** The total count of signals in the provided datasets.

Dataset ID	Dataset Name	Dataset Size
DS 1	35-mer Adenosine	15
DS 2	31-mer Oestradiol	24
DS 3	35-mer Oestradiol	24

**Table 2:** The datasets and the fundamental elements of the sensors responsible for recording signals in each dataset.

Dataset Name	Analyte	Transducer	Bioreceptor	Ref.
35-mer Adenosine	Adenosine	CNT FET	$5^{\prime }$-NH2-AAAAAAAAAACCTGGGGGAGTATTGCGGAGGAAGG-$3^{\prime }$	[29]
31-mer Oestradiol	Oestradiol	CNT FET	$5^{\prime }$-GGTCCTGACCGGAGGCTGACCGGAGTGGGAA-$3^{\prime }$	[30] ^1^
35-mer Oestradiol	Oestradiol	CNT FET	$5^{\prime }$-AAGGGATGCCGTTTGGGCCCAAGTTCGGCATAGTG-$3^{\prime }$	[31]

^1^ Erica S. Cassie created the aptamer for this dataset, which was a variation of the one described in the cited source. This sequence incorporated the shared segment of the best three oestradiol aptamers, with additional segments added at both ends.

**Table 3:** Comparison of the sensing procedures of the sensors. Note that the details regarding the oestradiol sensors are combined into a single column, as they share similar sensing procedures.

Characteristics	Adenosine Sensor	Oestradil Sensors
Time interval measurement	1 s	1.081 s with std $5\times 10^{-3}$
Gate voltage ($V_{G}$)	0 V	0 V
Drain voltage ($V_{D}$)	100 mV	100 mV
Buffer solution	2 mM Tris-HCI	0.05× PBS with 5% EtOH
Initial step load chemical	110 μM of 2 mM Tris-HCI	100 μL of 0.05× PBS 5% EtOH
Next steps load chemical	-	20 μL of 0.05× PBS 5% EtOH
Initial analyte load time	1000 s	600 s
Time interval of analyte injection	500 s	300 s
Time interval of chemical injection	-	300 s
Variation of analyte concentration	100 pM–10 μM	1 nM–10 μM

**Table 4:** The total number of segments corresponds to the datasets and is categorized based on their analyte concentration levels. It should be noted that the 100 pM segment in DS 1 was removed due to data scarcity and will not be mentioned in this study afterwards.

Analyte Concentration	DS 1	DS 2	DS 3
No Analyte	15	24	24
100 pM	1	0	0
1 nM	5	24	24
10 nM	7	24	24
100 nM	9	24	24
1 μM	12	24	24
10 μM	15	24	24
Total Segments	63	144	144

**Table 5:** The total size of normal segments for available datasets after applying anomaly detection.

Analyte Concentration	DS 1	DS 2	DS 3
No Analyte	9	18	5
1 nM	4	18	5
10 nM	4	18	11
100 nM	4	18	11
1 μM	4	18	11
10 μM	6	18	11

**Table 6:** Segment labeling for classification models C1 and C2.

Analyte Concentration	C1 Labels			C2 Labels		
	DS 1	DS 2	DS 3	DS 1	DS 2	DS 3
No Analyte	1	1	1	1	1	1
1 nM	2	2	2	2	7	7
10 nM	3	3	3	3	8	8
100 nM	4	4	4	4	9	9
1 μM	5	5	5	5	10	10
10 μM	6	6	6	6	11	11
Anomaly	-	-	-	12	12	12

**Table 7:** Layer description of the recurrent-based networks developed for signal extrapolation.

Layer Number	Layer Type	Hyperparameters	Learnable Parameters	State Parameters
1	Sequential input	Output size: 1	-	-
2	GRU	Input size: 1	$W_{x}$: 3n×1	Hidden state: *n* × 1
		Hidden units: *n*	$W_{h}$: 3n×n	
		Output size: *n*	*b*: 3n×1	
	ULSTM	Input size: 1	$W_{x}$: 4n×1	Hidden state: n×1
		Hidden units: *n*	$W_{h}$: 4n×n	
		Output size: *n*	*b*: 4n×1	Cell state: n×1
	BLSTM	Input size: 1	$W_{x}$: 8n×1	Hidden state: 2n×1
		Hidden units: *n*	$W_{h}$: 8n×n	
		Output size: 2n	*b*: 8n×1	Cell state: 2n×1
3	FC (GRU, ULSTM) ^1^	Input size: *n*	Weights: 1×n	-
		Output size: 1	Bias: 1×1	
	FC (BLSTM) ^2^	Input size: 2n	Weights: 1×2n	-
		Output size: 1	Bias: 1×1	
4	Regression output	Output size: 1	-	-

^1,2^ A fully connected layer that succeeds GRU, ULSTM, and BLSTM layers, respectively.

**Table 8:** Layer description and comparison of the developed recurrent-based networks for classification models.

Layer Number	Layer Type	Hyperparameters (Output Size)	Learnable Parameters	State Parameters
1	Sequential input	1	-	-
2	GRU	Input size: 1	$W_{x}$: 3n×1	
		Hidden units: *n*	$W_{h}$: 3n×n	Hidden state: n×1
		Output size: *n*	*b*: 3n×1	
	ULSTM	Input size: 1	$W_{x}$: 4n×1	Hidden state: n×1
		Hidden units: *n*	$W_{h}$: 4n×n	
		Output size: *n*	*b*: 4n×1	Cell state: n×1
	BLSTM	Input size: 1	$W_{x}$: 8n×1	Hidden state: 2n×1
		Hidden units: *n*	$W_{h}$: 8n×n	
		Output size: 2n	*b*: 8n×1	Cell state: 2n×1
3	FC (GRU, ULSTM)	*m*	Weights: m×n	-
			Bias: m×1	
	FC (BLSTM)	*m*	Weights: m×2n	-
			Bias: m×1	
4, 5	Softmax, Classification	*m*	-	-

**Table 9:** Architecture and layer descriptions of the proposed Conv-GRU, Conv-ULSTM, and Conv-BLSTM networks for classification models.

Layer Number	Layer Type	Hyperparameters (Output Size)	Learnable Parameters	State Parameters
1	Image Input	$l_{s}$	-	-
2	2D Convolution	$\left[l_{c}\;n_{f}\right]$	Weights: $s_{f}\times n_{f}$	-
			Bias: $1\times n_{f}$	
3	Leaky ReLU, scale = 0.1	$\left[l_{c}\;n_{f}\right]$	-	-
4	Flatten	$k=l_{c}\times n_{f}$	-	-
5	GRU	Input size: k	$W_{x}$: 3n×k	
		Hidden units: *n*	$W_{h}$: 3n×n	Hidden state: n×1
		Output size: *n*	*b*: 3n×1	
	ULSTM	Input size: k	$W_{x}$: 4n×k	Hidden state: n×1
		Hidden units: *n*	$W_{h}$: 4n×n	
		Output size: *n*	*b*: 4n×1	Cell state: n×1
	BLSTM	Input size: k	$W_{x}$: 8n×k	Hidden state: 2n×1
		Hidden units: *n*	$W_{h}$: 8n×n	
		Output size: 2n	*b*: 8n×1	Cell state: 2n×1
6	Leaky ReLU, scale = 0.1	GRU, ULSTM: *n*	-	-
		BLSTM: 2n		
7	FC (GRU, ULSTM)	*m*	Weights: m×n	-
			Bias: m×1	
	FC (BLSTM)	*m*	Weights: m×2n	-
			Bias: m×1	
8, 9	Softmax, Classification	*m*	-	-

**Table 10:** Architecture and layer descriptions of the proposed CNN network for classification models. Note that ConvBlock refers to a combination of a 2D convolution layer, a Leaky ReLU activation function, and a dropout layer placed sequentially.

Layer Number	Layer Type	Hyperparameters (Output Size)	Learnable Parameters
1	Image Input	$l_{s}$	-
2	2D Convolution	$\left[l_{c_{1}}\;n_{f_{1}}\right]$	Weights: $s_{f}\times n_{f_{1}}$
			Bias: $1\times n_{f_{1}}$
3	Leaky ReLU, scale = 0.1	$\left[l_{c_{1}}\;n_{f_{1}}\right]$	-
4	Dropout, probability = 0.25	$\left[l_{c_{1}}\;n_{f_{1}}\right]$	-
5, 6, 7	2D ConvBlock	$\left[l_{c_{2}}\;n_{f_{2}}\right]$	Weights: $s_{f}\times n_{f_{2}}$
			Bias: $1\times n_{f_{2}}$
8, 9, 10	2D ConvBlock	$\left[l_{c_{3}}\;n_{f_{3}}\right]$	Weights: $s_{f}\times n_{f_{3}}$
			Bias: $1\times n_{f_{3}}$
11, 12, 13	2D ConvBlock	$\left[l_{c_{4}}\;n_{f_{3}}\right]$	Weights: $s_{f}\times n_{f_{3}}$
			Bias: $1\times n_{f_{3}}$
14, 15, 16	2D ConvBlock	$\left[l_{c_{5}}\;n_{f_{2}}\right]$	Weights: $s_{f}\times n_{f_{2}}$
			Bias: $1\times n_{f_{2}}$
17, 18, 19	2D ConvBlock	$\left[l_{c_{6}}\;n_{f_{1}}\right]$	Weights: $s_{f}\times n_{f_{1}}$
			Bias: $1\times n_{f_{1}}$
20	FC	Input size: $k=l_{c_{6}}\times n_{f_{1}}$	Weights: m×k
		Output size: *m*	Bias: m×1
21, 22	Softmax, Classification	*m*	-

**Table 11:** Detailed information regarding the output sizes of the layers in the networks developed for data augmentation. Additionally, “S”, “C”, and “B” denote the spatial size, channel number, and batch size, respectively. Furthermore, CVAE and VAE represent the networks used for data augmentation of normal and anomaly data, respectively.

Layers		Layers Name	Encoder		Decoder	
**CVAE**	**VAE**	**Encoder/Decoder**	**DS 1**	**DS 2 & DS 3**	**DS 1**	**DS 2 & DS 3**
1	1	Image/Feature Input	500 (S) × 1 (S) × 1 (C) × 1 (B)	300 (S) × 1 (S) × 1 (C) × 1 (B)	32 (C) × 1 (B)	32 (C) × 1 (B)
2	2	FC	500 (C) × 1 (B)	300 (C) × 1 (B)	4096 (C) × 1 (B)	2048 (C) × 1 (B)
3	3	Reshape	500 (S) × 1 (S) × 1 (C) × 1 (B)	300 (S) × 1 (S) × 1 (C) × 1 (B)	64 (S) × 1 (S) × 64 (C) × 1 (B)	32 (S) × 1 (S) × 64 (C) × 1 (B)
4	-	Feature Input	1 (C) × 1 (B)	1 (C) × 1 (B)	1 (C) × 1 (B)	1 (C) × 1 (B)
5	-	Embedding	32 (C) × 1 (B)	32 (C) × 1 (B)	32 (C) × 1 (B)	32 (C) × 1 (B)
6	-	FC	500 (C) × 1 (B)	300 (C) × 1 (B)	64 (C) × 1 (B)	32 (C) × 1 (B)
7	-	Reshape	500 (S) × 1 (S) × 1 (C) × 1 (B)	300 (S) × 1 (S) × 2 (C) × 1 (B)	64 (S) × 1 (S) × 1 (C) × 1 (B)	32 (S) × 1 (S) × 1 (C) × 1 (B)
8	-	Concatenation	500 (S) × 1 (S) × 2 (C) × 1 (B)	300 (S) × 1 (S) × 2 (C) × 1 (B)	64 (S) × 1 (S) × 65 (C) × 1 (B)	32 (S) × 1 (S) × 65 (C) × 1 (B)
9	4	Conv/Deconv	250 (S) × 1 (S) × 16 (C) × 1 (B)	150 (S) × 1 (S) × 16 (C) × 1 (B)	63 (S) × 1 (S) × 16 (C) × 1 (B)	38 (S) × 1 (S) × 16 (C) × 1 (B)
10	5	Leaky ReLU, scale = 0.1	250 (S) × 1 (S) × 16 (C) × 1 (B)	150 (S) × 1 (S) × 16 (C) × 1 (B)	63 (S) × 1 (S) × 16 (C) × 1 (B)	38 (S) × 1 (S) × 16 (C) × 1 (B)
11	6	Dropout, probability = 0.25	250 (S) × 1 (S) × 16 (C) × 1 (B)	150 (S) × 1 (S) × 16 (C) × 1 (B)	63 (S) × 1 (S) × 16 (C) × 1 (B)	38 (S) × 1 (S) × 16 (C) × 1 (B)
12	7	Conv/Deconv	125 (S) × 1 (S) × 16 (C) × 1 (B)	75 (S) × 1 (S) × 16 (C) × 1 (B)	125 (S) × 1 (S) × 16 (C) × 1 (B)	75 (S) × 1 (S) × 16 (C) × 1 (B)
13	-	Conv/Deconv	125 (S) × 1 (S) × 16 (C) × 1 (B)	75 (S) × 1 (S) × 16 (C) × 1 (B)	63 (S) × 1 (S) × 16 (C) × 1 (B)	75 (S) × 1 (S) × 16 (C) × 1 (B)
14	-	Addition	125 (S) × 1 (S) × 16 (C) × 1 (B)	75 (S) × 1 (S) × 16 (C) × 1 (B)	63 (S) × 1 (S) × 16 (C) × 1 (B)	75 (S) × 1 (S) × 16 (C) × 1 (B)
15	8	Leaky ReLU, scale = 0.1	125 (S) × 1 (S) × 16 (C) × 1 (B)	75 (S) × 1 (S) × 16 (C) × 1 (B)	63 (S) × 1 (S) × 16 (C) × 1 (B)	75 (S) × 1 (S) × 16 (C) × 1 (B)
16	9	Dropout, probability = 0.25	125 (S) × 1 (S) × 16 (C) × 1 (B)	75 (S) × 1 (S) × 16 (C) × 1 (B)	63 (S) × 1 (S) × 16 (C) × 1 (B)	75 (S) × 1 (S) × 16 (C) × 1 (B)
17	10	Conv/Deconv	63 (S) × 1 (S) × 16 (C) × 1 (B)	38 (S) × 1 (S) × 16 (C) × 1 (B)	249 (S) × 1 (S) × 16 (C) × 1 (B)	150 (S) × 1 (S) × 16 (C) × 1 (B)
18	11	Conv/Deconv	63 (S) × 1 (S) × 16 (C) × 1 (B)	38 (S) × 1 (S) × 16 (C) × 1 (B)	249 (S) × 1 (S) × 16 (C) × 1 (B)	150 (S) × 1 (S) × 16 (C) × 1 (B)
19	12	Addition	63 (S) × 1 (S) × 16 (C) × 1 (B)	38 (S) × 1 (S) × 16 (C) × 1 (B)	249 (S) × 1 (S) × 16 (C) × 1 (B)	150 (S) × 1 (S) × 16 (C) × 1 (B)
20	13	Leaky ReLU, scale = 0.1	63 (S) × 1 (S) × 16 (C) × 1 (B)	38 (S) × 1 (S) × 16 (C) × 1 (B)	249 (S) × 1 (S) × 16 (C) × 1 (B)	150 (S) × 1 (S) × 16 (C) × 1 (B)
21	14	Dropout, probability = 0.25	63 (S) × 1 (S) × 16 (C) × 1 (B)	38 (S) × 1 (S) × 16 (C) × 1 (B)	249 (S) × 1 (S) × 16 (C) × 1 (B)	150 (S) × 1 (S) × 16 (C) × 1 (B)
22	15	FC/Deconv	64 (C) × 1 (B)	64 (C) × 1 (B)	500 (S) × 1 (S) × 1 (C) × 1 (B)	300 (S) × 1 (S) × 1 (C) × 1 (B)
			*Z*: 32 (C) × 1 (B)	32 (C) × 1 (B)		
23	16	Sampling	$\mu_{z}$: 32 (C) × 1 (B)	32 (C) × 1 (B)	500 (S) × 1 (S) × 1 (C) × 1 (B)	300 (S) × 1 (S) × 1 (C) × 1 (B)
			$\sigma_{z}$: 32 (C) × 1 (B)	32 (C) × 1 (B)		

**Table 12:** The performance metrics of the proposed data augmentation method and examples of data generated by the decoder modules of the CVAEs are presented. Columns 2 and 4 show examples of reconstructed and augmented segments, respectively, corresponding to their respective datasets. The segments for reconstruction were randomly selected from the test data and augmented data. AC refers to the analyte concentration of the segments.

Dataset	Reconstructed Segment	Reconstruction Error	Generated Data
35mer Adenosine	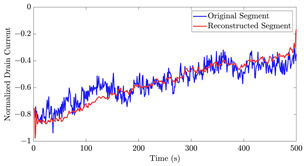 AC: no analyte	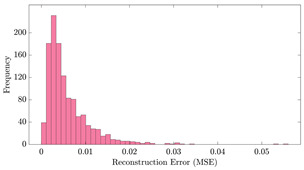	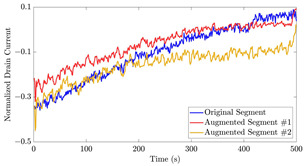 AC: 1 nM
31mer Oestradiol	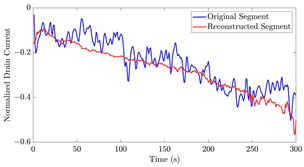 AC: 10 nM	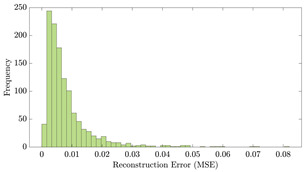	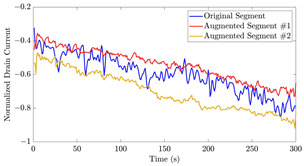 AC: 100 nM
35mer Oestradiol	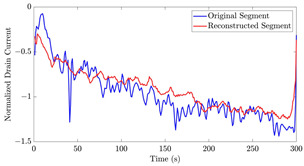 AC: 1 μM	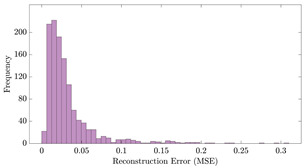	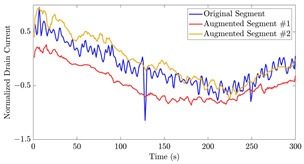 AC: 10 μM

## Data Availability

The data presented in this study might be available on request from the corresponding author. There are restrictions on data availability due to their necessity for our future work.

## Abbreviations

The following abbreviations are used in this manuscript:

ML	Machine Learning
DL	Deep Learning
STEM	Scanning Transmission Electron Microscopy
IDS	Intrusion Detection System
AC	Analyte Concentrations
CNT	Carbon Nanotube
STFT	Short-Term Fourier Transform
FET	Field-Effect Transistor
GAN	Generative Adversarial Network
AE	Autoencoder
VAE	Variational Autoencoder
CVAE	Conditional Variational Autoencoder
CNN	Convolution Neural Network
RNN	Recurrent Neural Networks
LSTM	Long Short-Term Memory
ULSTM	Unidirectional Long Short-Term Memory
BLSTM	Bidirectional Long Short-Term Memory
GRU	Gated Recurrent Unit
FC	Fully Connected
ELBO	Evidence Lower Bound
AEVB	Autoencoding Variational Bayes
SGVB	Stochastic Gradient Variational Bayes
KL	Kullback–Leibler
